# Rationally designed bacterial consortia to treat chronic immune-mediated colitis and restore intestinal homeostasis

**DOI:** 10.1038/s41467-021-23460-x

**Published:** 2021-05-28

**Authors:** Daniel van der Lelie, Akihiko Oka, Safiyh Taghavi, Junji Umeno, Ting-Jia Fan, Katherine E. Merrell, Sarah D. Watson, Lisa Ouellette, Bo Liu, Muyiwa Awoniyi, Yunjia Lai, Liang Chi, Kun Lu, Christopher S. Henry, R. Balfour Sartor

**Affiliations:** 1 Gusto Global LLC, Morrisville, NC USA; 2 Departments of Medicine, Microbiology and Immunology, Center for Gastrointestinal Biology and Disease, University of North Carolina at Chapel Hill, Chapel Hill, NC USA; 3 Department of Internal Medicine II, Shimane University Faculty of Medicine, Shimane, Japan; 4 Department of Medicine and Clinical Science, Graduate School of Medical Sciences, Kyushu University, Fukuoka, Fukuoka Japan; 5 Department of Environmental Sciences and Engineering, Gillings School of Public Health, University of North Carolina at Chapel Hill, Chapel Hill, NC USA

**Keywords:** Microbiology, Gastroenterology

## Abstract

Environmental factors, mucosal permeability and defective immunoregulation drive overactive immunity to a subset of resident intestinal bacteria that mediate multiple inflammatory conditions. GUT-103 and GUT-108, live biotherapeutic products rationally designed to complement missing or underrepresented functions in the dysbiotic microbiome of IBD patients, address upstream targets, rather than targeting a single cytokine to block downstream inflammation responses. GUT-103, composed of 17 strains that synergistically provide protective and sustained engraftment in the IBD inflammatory environment, prevented and treated chronic immune-mediated colitis. Therapeutic application of GUT-108 reversed established colitis in a humanized chronic T cell-mediated mouse model. It decreased pathobionts while expanding resident protective bacteria; produced metabolites promoting mucosal healing and immunoregulatory responses; decreased inflammatory cytokines and Th-1 and Th-17 cells; and induced interleukin-10-producing colonic regulatory cells, and IL-10-independent homeostatic pathways. We propose GUT-108 for treating and preventing relapse for IBD and other inflammatory conditions characterized by unbalanced microbiota and mucosal permeability.

## Abstract (web summary)

Fecal microbiota transplantation and probiotics have been tested/used as potential therapeutics against inflammatory bowel diseases (IBD). Here the authors use a bottom-up rational consortium design approach that combines well-characterized strains isolated from healthy human stool samples to produce two consortia of metabolically interdependent strains for the treatment of IBD.

## Introduction

Chronic intestinal inflammation can be induced by multiple exogenous and endogenous signals and is mediated by immune and nonimmune cells in genetically susceptible hosts with defects in epithelial barrier function, immunoregulation, or bacterial killing. Exogenous substances including dietary products, pathogenic microorganisms, xenobiotics including antibiotics, or various combinations thereof, can trigger initial mucosal injury and/or dysbiosis to initiate acute intestinal inflammation that is perpetuated by the antigenic activities of a subset of resident microbiota^1,2^. Examples of these conditions are inflammatory bowel diseases (IBD), which encompass two main clinical disorders: Crohn’s disease and ulcerative colitis. Current IBD treatments primarily control inflammation through anti-inflammatory and immunosuppressive mechanisms. Some of the most successful drugs for treating IBD include infliximab, adalimumab, vedolizumab, ustekinumab, and tofacitinib, which target specific immune components to control the inflammatory process. However, these and other immune modulating drugs induce sustained, steroid-free remission in only a small subset of patients and can have multiple serious side effects, including an increased risk for serious and potentially life-threatening infections and neoplasia. In addition, these drugs do not correct upstream conditions that contribute to the chronic inflammatory mechanisms, including the leaky mucosal barrier, a pro-inflammatory gut microbiome and immunoregulatory defects.

As an alternative to anti-inflammatory and immunosuppressive therapies, microbiome-inspired live biotherapeutic products (LBPs) are being developed to treat conditions linked to chronic intestinal inflammation and increased permeability. The traditional approach for LBP discovery has been to compare the microbiomes of healthy subjects and patients suffering from a specific condition, such as IBD, to identify microorganisms that are lacking or under-represented in large databases, such as the HMP2 project^3^. This information, further enforced by the results from microbial association studies, is used to propose a therapeutic formulation to replenish the microorganisms that are lacking or under-represented^2,4^. In the case of IBD, early efforts have focused on the use of strains belonging to the *Clostridium* clusters IV and XIVa, which were found to successfully decrease inflammation in rodent IBD models^5–7^. Using germ-free (GF) mice inoculated with healthy human fecal material pretreated with chloroform to enrich for spore-forming bacteria, a stable 17-strain consortium was enriched from a single donor based on their ability to induce colonic regulatory T cells (Tregs)^7,8^. This consortium was comprised of spore-forming *Clostridium* cluster IV, XIVa, and XVIII strains that produced butyrate and decreased the severity of several colitis models^7^.

Open label application of Fecal Microbiome Transplants and enrichment-based approaches have several disadvantages. The outcome is defined by the stool sample used for the enrichment, with different samples representing different consortia with variable efficacy;^9^ undesirable strains/functions associated with safety risks including virulence factors and transferable antibiotic resistance functions might be present, such as the presence of enteropathogenic and Shigatoxin-producing *Escherichia coli* strains^10^ and antibiotic-resistant *E. coli* strains^11^ in FMTs, or the presence of transferable vancomycin resistance elements as found in the genome of the VE202 consortium strain *Blautia coccoides* VE202-06 (GenBank Accession Number Accession: PRJDB525). Furthermore, consortium modeling, as presented in this study, shows that other bacterial species besides spore-forming *Clostridium* bacteria provide metabolic support and additional therapeutic functions required for optimal engraftment and therapeutic performance of live biotherapeutic products in the hostile gut environment of patients with intestinal inflammation. These shortcomings can be addressed by a bottom-up rational consortium design approach that is rigorously informed by mechanistic modeling and insights from microbiome ecology and disease pathogenesis. We used this approach to combine well-characterized strains isolated from many healthy human stool samples into a consortium of metabolically interdependent strains with a variety of therapeutic functionalities being distributed in a redundant way between strains. Initially a 17-strain consortium, GUT-103, was designed around publicly available strains. GUT-103 rapidly colonized mice, restored normal function to the inflamed colon, and prevented and reversed established experimental colitis in gnotobiotic mice. Based on these proof of concept studies, a refined 11-member consortium, GUT-108, was designed around a panel of proprietary human bacterial strains that strongly engrafted and provided similar redundant protective functions. Therapeutically applied GUT-108 corrected functional dysbiosis of the inflamed gut microbiome and treated established colitis in a humanized mouse colitis model while decreasing opportunistic pathogenic bacteria, increasing resident protective bacterial groups, and restoring immunologic and metabolic homeostasis.

## Results

### Rational design of the GUT-103 consortium

Using a bottom-up approach, LBPs were rationally designed to provide key therapeutic functions involved in mucosal homeostasis and immune modulation defective in the gut microbiome of IBD patients^2,3^. These functions include synthesis of short-chain fatty acids (SCFA) including butyrate and propionate, synthesis of indole, indole-3 propionic acid (IPA) and indole-3 acetic acid (IAA) from tryptophan, and deconjugation of bile salts and their conversion into therapeutic secondary bile acids, especially deoxycholic acid (DCA) and lithocholic acid (LCA). Additional functions included to control pathobionts were the synthesis of antimicrobials and siderophores and the uptake of heterologous siderophores produced by *Enterobacteriaceae* including *Escherichia, Klebsiella*, and *Shigella* species, allowing bacteria to compete with these opportunistic pathogens for the essential nutrient iron. The importance of the gut microbiome to provide these specific functions^12–14^ and the role of specific microbes encoding key activities, such as the 7α/β-dehydroxylation bile acid conversion pathway encoded by *Clostridium scindens*^15^ has been extensively reviewed.

Next, commensal human gut bacteria that can provide these desired functions were identified. Reference isolate genome sequences for common human gut microbes were collected based on published gut 16 S rRNA studies and isolate source metadata (https://www.vmh.life/#home). Isolate and metagenome assembled genome (MAG) sequences were gathered from RefSeq^16^, PATRIC^17^ and the Human Microbiome Project^18^. Consistently annotated genomes were used to determine which genomes were likely to encode proteins with the desired therapeutic functions. Comparative analysis of annotations from these genomes also enabled the identification of genera where selected therapeutic functions were highly conserved. A key finding from this analysis was that members of the *Clostridium* clusters IV and XIVa families could not provide several critical functionalities required to restore the pro-inflammatory microbiome of IBD patients, including the synthesis of propionate and indole-based derivatives from tryptophan. Therefore, the rational design of LBPs to restore these critical functionalities should also include members of the families *Bacteroidaceae, Eubacteriaceae, Akkermansiaceae*, and non-*Clostridium* clusters IV and XIVa family members. Ultimately, a set of the most desirable genera were selected from which to compose the rationally designed LBPs, including isolates belonging to the genera *Akkermansia* (Supplementary Data 1), *Bacteroides* (Supplementary Data 2), *Clostridium* (Supplementary Data 3), and *Faecalibacterium* (Supplementary Data 4). Multi-genome analysis for the genera *Akkermansia* and *Faecalibacterium* combined with whole-genome DNA average nucleotide identity (ANI) showed that species previously classified as *Akkermansia muciniphila* and *Faecalibacterium prausnitzii* are comprised of several species, distinguished by over 10% difference in whole-genome DNA sequence compared to their type strains.

Although this comparative analysis identified desirable genera, it was still necessary to identify specific strains within each genus to comprise the consortia. In this selection process, it is most important to identify strains with complementary auxotrophies for essential amino acids, vitamins, and co-factors. This was based on previous analyses of natural microbiomes, which revealed that co-evolved strains in these systems often have complementary auxotrophies^19^, and addresses the risk of one strain outcompeting the other members of the consortium. Furthermore, redundancy in metabolic dependencies between strains is important to avoid that loss of one strain will not lead to the collapse of the consortium. Auxotrophic analysis of isolate strain genomes and MAGs from our selected genera revealed that auxotrophies were typically conserved among most members of the same species, but with enough variation to make this analysis important to include in the design process.

Overall, using redundancy in key functionalities combined with a distribution of complementary auxotrophies to create a network of metabolically interdependent strains, a consortium of 17 publicly available human strains was selected, referred to as GUT-103 (Table 1). For each of the strains, individual strain models were constructed that were subsequently combined into a consortium model. Flux Balance analysis was used to predict in silico that the GUT-103 strains form a network of metabolic dependencies complementing each other’s metabolic needs, indicating that GUT-103 can function as a consortium without the need for auxotrophies being complemented by resident bacteria from the imbalanced gut microbiome of IBD patients^3^. The GUT-103 functional characteristics, auxotrophy reactions, flux balance analysis, and strain interaction table to predict the growth of the individual GUT-103 strains and the GUT-103 consortium under defined conditions are shown in Supplementary Data 5.

**Table 1:** Overview of GUT-103 strains and their key therapeutic properties, including the synthesis of butyrate, propionate and indole.

Strain			Functions under-represented for IBD					
Species	Family	PATRIC ID (genome sequence)	Butyrate	Propionate	Indole	Siderophore	Bile Salt	Antimicrobial
*Megamonas funiformis* DSM19343	Selenomonadaceae	742816.3		+		Ferrichrome and Enterobactin uptake		
*Megamonas hypermegale* DSM1672	Selenomonadaceae	1122216.3		+		Ferrichrome uptake		
*Acidaminococcus intestini* DSM21505	Acidaminococcaceae	1120921.3	+					
*Bacteroides massiliensis* DSM17679	Bacteroidaceae	1121098.3		+		Heterologous uptake		
*Bacteroides stercoris* ATCC43183 / DSM19555	Bacteroidaceae	449673.7		+	+	Heterologous incl. Enterobactin uptake		
*Barnesiella intestinihominis* DSM21032	Porphyromonadaceae	742726.3		+		Heterologous incl. Aerobactin uptake		
*Faecalibacterium prausnitzii* DSM17677	Ruminococcaceae	411483.3	+			Heterologous uptake		Bacteriocin
*Subdoligranulum variabile* DSM15176	Ruminococcaceae	411471.5	+					Bacteriocin
*Anaerostipes caccae* DSM14662	Lachnospiraceae	411490.6	+			Heterologous uptake incl. Ferrichrome; Yersiniabactin synthesis	7-α-HSD	Bacteriocin
*Anaerostipes hadrus* DSM3319 / ATCC 29173	Lachnospiraceae	649757.3	+		+			
*Clostridium symbiosum* ATCC14940	Lachnospiraceae	411472.5	+				3-α-HSD, 7-α-HSD	
*Akkermansia muciniphila* ATCC BAA-835	Akkermansiaceae	349741.6		+	+	Heterologous uptake		
*Clostridium scindens* ATCC35704	Lachnospiraceae	411468.9					7-α-DH	Bacteriocin
*Clostridium bolteae* ATCC BAA-613*	Lachnospiraceae	411902.9				Siderophore synthesis	3-α-HSD, 7-α-HSD	Bacteriocin
*Blautia producta* DSM2950*	Lachnospiraceae	1121114.4	+			Heterologous incl. Ferrichrome uptake		
*Blautia hydrogenotrophia* DSM10507*	Lachnospiraceae	476272.21					7-α-DH, 3-β-HSD	Bacteriocin
*Marvinbryantia formatexigens* DSM14469*	Lachnospiraceae	478749.5				Heterologous incl. Ferrichrome uptake		

Functions involved in the synthesis of siderophores, the uptake of heterologous siderophores, and the deconjugation of bile salts and the conversion to secondary bile acids are indicated (+).

*3α-HSD* 3α-hydroxy steroid dehydrogenase, *7α-HSD* 7α-hydroxy steroid dehydrogenase, *7β -HSD* 7β-hydroxy steroid dehydrogenase, *7α-DH* 7α-dehydratase.

### GUT-103 successfully prevents and treats experimental colitis

**Fig. 1:**
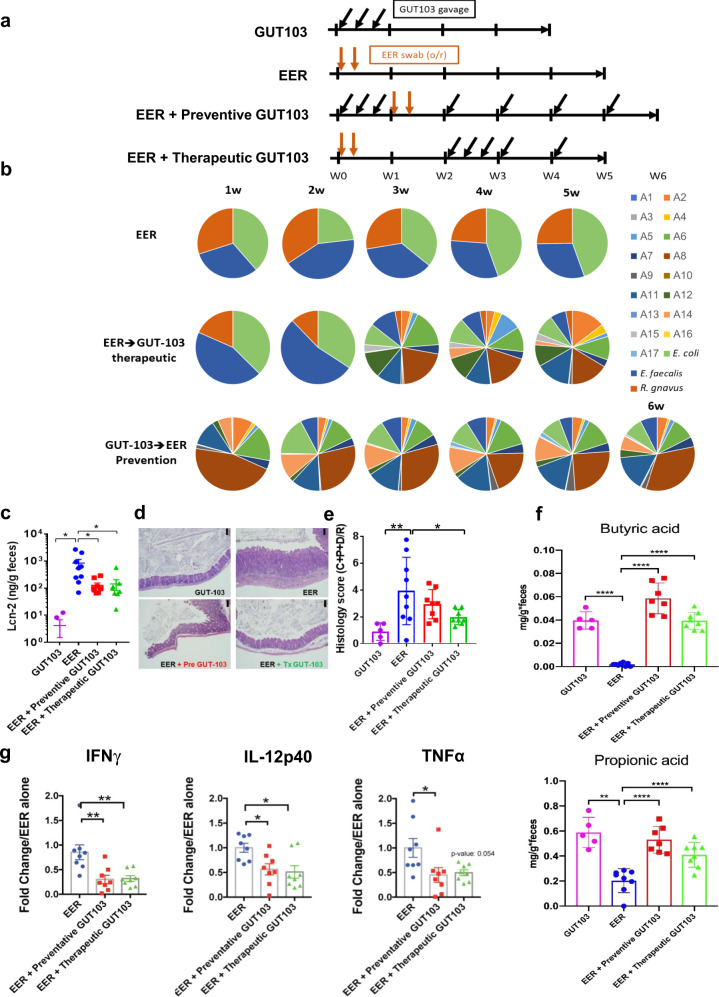
Effect of gavage with GUT-103 on the gut microbiome community composition, lipocalin-2 levels, histology of the gut epithelium, SCFA synthesis and tissue cytokine expression of *Il10*^*−/−*^ mice where experimental colitis was induced by inoculation with *Escherichia coli* LF82 (E), *Enterococcus faecalis* OG1RF (E) and *Ruminococcus gnavus* ATCC29149 (R). **a** Schematic overview of experimental design. Gnotobiotic *Il10*^*−/−*^ mice were inoculated with GUT-103 (*N* = 5), EER (*N* = 9), GUT-103 plus EER (preventive protocol) (N-7), and EER plus GUT-103 (therapeutic protocol) (*N* = 7). After 5 or 6 weeks, animals were killed and tissue samples were collected for analysis; **b** Composition of the microbiome in fecal pellets of gnotobiotic 129 *Il10*^*−/−*^ mice inoculated with EER, GUT-103 plus EER (preventive protocol), and EER plus GUT-103 (therapeutic protocol) determined by qPCR. *Il10*^*−/−*^ mice were inoculated via gavage with GUT-103 (2 × 10^+7^ cfu/strain) or EER (2 × 10^+7^ cfu/strain). The composition of the gut microbiome was determined on fecal pellets or after sacrifice on cecal contents using qPCR with species-specific primers. The average community composition (pie diagrams) for seven animals per treatment are presented. Strain legend: A1: *Megamonas hypermegale* DSM1672; A2 *Bacteroides stercoris* DSM19555; A3: *Anaerostipes hadrus* DSM3319; A4: *Clostridium symbiosum* ATCC14940; A5: *Clostridium boltea* ATCC BAA-613; A6: *Blautia producta* DSM2950; A7: *Clostridium scindens* ATCC35704; A8: *Akkermansia muciniphila* ATCC BAA-835; A9: *Megamonas funiformis* DSM19343; A10: *Acidaminococcus intestini* DSM21505; A11: *Bacteroides massiliensis* DSM17679; A12: *Barnesiella intestinihominis* DSM21032; A13: *Faecalibacterium prausnitzii* DSM17677; A14: *Subdoligranulum variabile* DSM15176; A15: *Anaerostipes caccae* DSM14662; A16: *Blautia hydrogenotrophica* DSM10507; A17: *Marvinbryantia formatexigens* DSM14469. **c** Cecal content lipocalin-2 levels at time of sacrifice from GUT-103 group (*N* = 5), EER group (*N* = 9), GUT-103 plus EER group (*N* = 9), and EER plus GUT-103 group (*N* = 8), Mann–Whitney unpaired two-tailed *t*-test. Mean ± SE, *P < 0.05. **d** Representative histology pictures of H&E-stained cecum tissue (×100). The bar (upper right corner) in the pictures indicates 100 µm from GUT-103 group (experiment was repeated five times independently with similar results), EER group (experiment was repeated nine times independently with similar results), GUT-103 plus EER group (experiment was repeated eight times independently with similar results), and EER plus GUT-103 group (experiment was repeated eight times independently with similar results). **e** Histology scores of GUT-103 group (*N* = 5), EER group (*N* = 9), GUT-103 plus EER group (*N* = 8), and EER plus GUT-103 group (*N* = 8). Combined score of cecum, proximal colon, and distal colon/rectum. 1-way ANOVA, Mean ± SE, *P < 0.05, **P < 0.01. **f** Fecal metabolite profiles for butyrate and propionate in *Il10*^*−/−*^ mice inoculated with GUT-103 (*N* = 5), GUT-103 plus EER (preventive protocol, *N* = 8), and EER plus GUT-103 (therapeutic protocol, *N* = 8) compared to EER (*N* = 8). Mann–Whitney unpaired two-tailed *t*-test. ±SE, **P < 0.01, ****P < 0.001. **g** Cecal tissue levels of cytokine mRNA expression of GUT-103 (*N* = 5), EER (*N* = 8), GUT-103 plus EER (preventive protocol, *N* = 8), and EER plus GUT-103 (therapeutic protocol, *N* = 8). Samples were collected at necropsy. One-way ANOVA, Mean ± SE, *P < 0.05, **P < 0.01. Source data and calculated *P*-values are provided as a Source Data file.

Preliminary testing using quantitative PCR with species-specific *rpoB* gene primers showed that 2 weeks after gavage GUT-103 successfully colonized GF 129 WT and 129 interleukin-10 deficient (*Il10*^*−/−*^) mice (see Supplementary Fig. 1a, b and Supplementary Table 1). Three human bacteria, *Escherichia coli* LF82, *Enterococcus faecalis* OG1RF, and *Ruminococcus gnavus* ATCC29149 (EER strains) were used based on their ability to colonize GF 129 WT and 129 *Il10*^*−/−*^ mice (Supplementary Fig. 2a, b and Supplementary Table 2), to induce chronic colitis and to activate strain-specific Th1 and Th17 mucosal immune responses in gnotobiotic *Il10*^*−/−*^ mice^20,21^. Selective colonization of gnotobiotic *Il10*^*−/−*^ mice by EER showed increased fecal lipocalin-2 concentrations (149.8 ± 80.2 ng/g feces, a 2 log increase from baseline values) at 2 weeks, with further progression over 4 weeks (Supplementary Fig. 3). Based on these kinetic studies showing mildly active colitis 2 weeks after EER colonization, we chose 2 weeks for GUT-103 therapeutic intervention. The ability of GUT-103 to colonize, compete with IBD-relevant pathobionts and to both prevent and treat establish Th1/Th17-mediated colitis was subsequently tested (Fig. 1a). GUT-103 was successfully established one week after gavage in EER *Il10*^*−/−*^ mice, (Fig. 1b) with a composition similar to that in ex-GF mice (Supplementary Fig. 1). All strains were detected but concentrations varied. Three dominant strains, *Blautia producta* DSM2950, *Akkermansia muciniphila* ATCC BAA-835 and *Bacteroides massiliensis* DSM17679, with complementary auxotrophies, suggest the formation of an integrated metabolic network. Relatively high levels of *Bacteroides stercoris* DSM19555, which has similar auxotrophies and functions as *Bacteroides massiliensis* DSM17679, were also observed. These predominant community strains are predicted to synthesize SCFA and indole, but lack synthesis of siderophores, bacteriocin synthesis and bile acid conversion. GUT-103 decreased concentrations of each of the EER strains in aggregate to less than 20% of the total bacterial population, even in the therapeutic protocol where the three colitogenic strains were established for 2 weeks (Fig. 1b). The preventive protocol blocked *R. gnavus* concentrations to below 1%.

Colonization with GUT-103 did not induce inflammation, but both the preventive and therapeutic protocols significantly decreased colitis in EER-inoculated *Il10*^*−/−*^ mice compared with PBS controls, as measured by lipocalin-2 levels (Fig. 1c), crypt hyperplasia and lamina propria (LP) mononuclear cell infiltration (Fig. 1d), interaction table predict the growth of the individual GUT-108 and blinded histology scoring (Fig. 1e). Gavage with GUT-103 also increased synthesis of butyrate and propionate that was deficient in EER-inoculated *Il10*^*−/−*^ mice (Fig. 1f) and decreased inflammation measured by cecal mRNA expression for IFNγ, IL-12p40, and TNFα (Fig. 1g).

**Fig. 2:**
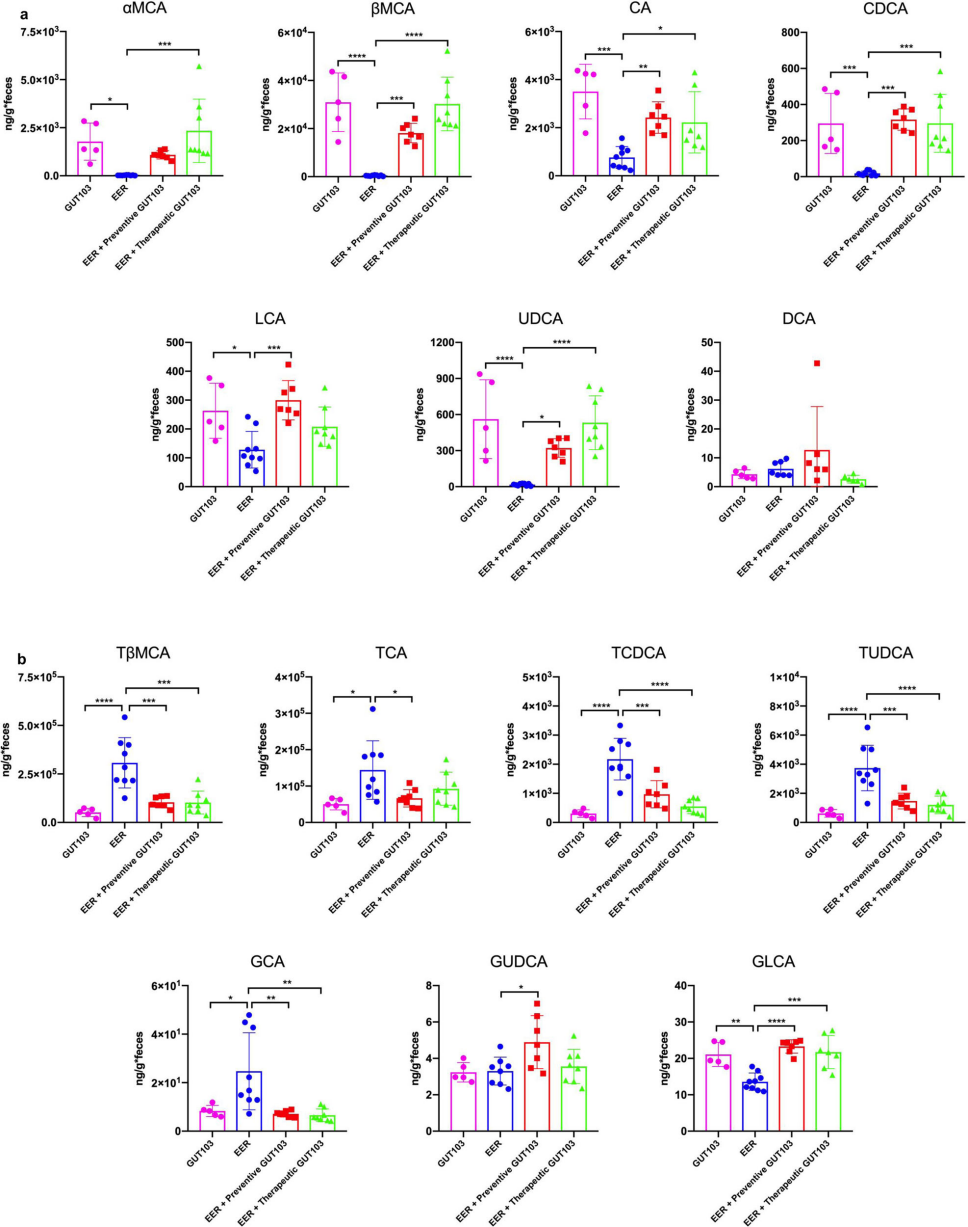
Levels of unconjugated and conjugated bile acids in the cecal content of *Il10*^−/−^ mice at the time of sacrifice where experimental colitis was induced by inoculation with *Escherichia coli* LF82 (E), *Enterococcus faecalis* OG1RF (E), and *Ruminococcus gnavus* ATCC29149 (R). Gnotobiotic *Il10*^*−/−*^ mice were inoculated with GUT-103 (*N* = 5), EER (*N* = 9), GUT-103 plus EER (preventive protocol) (*N* = 7), and EER plus GUT-103 (therapeutic protocol) (*N* = 7). *Il10*^*−/−*^ mice were inoculated via gavage with GUT-103 (2 × 10^+7^ cfu/strain) or EER (2 × 10^+7^ cfu/strain). **a** Unconjugated bile acids: αMCA α-muricholic acid, βMCA β-muricholic acid, CA cholic acid, CDCA chenodeoxycholic acid, LCA lithocholic acid, UDCA ursodeoxycholic acid, DCA deoxycholic acid. **b** Conjugated bile acids: TβMCA taurine-conjugated β-muricholic acid, TCA taurocholic acid, TCDCA taurochenodeoxycholic acid, TUDCA tauroursodeoxycholic acid, GUDCA glycoursodeoxycholic acid, GCA glycine-conjugated cholic acid, GLCA glycine-conjugated lithocholic acid. 1e-way ANOVA, Mean ± SE, *P < 0.05, **P < 0.01, ***P < 0.005, ****P < 0.001. Source data and calculated *P*-values are provided as a Source Data file.

Bile acid profiles were analyzed to further evaluate restoration of a functional gut microbiome by engraftment with GUT-103 strains. Significantly higher levels of multiple unconjugated bile acids were observed in cecal contents from Therapy, Prevention and GUT-103 groups compared with EER-inoculated *Il10*^*−/−*^ mice (Fig. 2a). Compared to EER, therapeutic application of GUT-103 increased cecal luminal concentrations of the unconjugated primary bile acids βMCA, CA, and CDCA. Cecal contents of the GUT-103 group had higher levels of the secondary bile acids UDCA and LCA than EER-inoculated *Il10*^*−/−*^ mice, but low DCA levels were not significantly different between these groups (Fig. 2a). In contrast to free bile acids, the cecal luminal levels of multiple conjugated bile acids, including TβMCA, TCDCA, TUDCA, and GCA, in all GUT-103 groups were significantly lower than those in EER (Fig. 2b). However, the level of glycine-conjugated GLCA was significantly higher in the GUT-103-colonized group than in the EER group (Fig. 2b). Overall, compared to EER, GUT-103 decreased levels of conjugated bile acids especially for taurine-conjugated bile acids. Together, these results demonstrate both protective and therapeutic activities of GUT-103 and in vivo validation of the anticipated replacement of pathobionts and protective metabolic functions of this rationally designed consortium. It should also be noted that in preliminary studies we demonstrated that two independent consortia of 14 and 19 bacterial isolates, respectively, from two healthy human volunteers induced colitis when colonized into germ-free 129 *Il10*^*−/−*^ mice^22,23^.

### Design of the optimized GUT-108 consortium

Informed by the GUT-103 outcomes, we rationally designed an optimized 11-strain consortium referred to as GUT-108 (Table 2). Compared to GUT-103, GUT-108 included improved redundancy for synthesis of the therapeutic secondary bile acids LCA and DCA covered by *Extibacter* sp. GGCC_0201; yersiniabactin synthesis provided by *Clostridium symbiosum* GGCC_0272; and several strains with functions antagonistic against opportunistic pathogens into the *Enterobacteriaceae* family. The GUT-108 functional characteristics, auxotrophy reactions, flux balance analysis, and strain interaction table predict the growth of the individual GUT-108 strains and the GUT-108 consortium under defined conditions and are shown in Supplementary Data 6.

**Table 2:** Overview of GUT-108 strains and their key therapeutic properties, including the synthesis of butyrate, propionate, gamma-amino-butyrate (GABA), and indole.

Strain	Family	Butyrate	Propionate	GABA	Indole	Bile Acid	Clostridium cluster	GENBANK ID	Additional information
*Bacteroides xylanisolvens* GGCC_0124	Bacteroidaceae		+	+	+	7-α-HSD, CGH, 3-oxo-5-α	N.A.	JABFCE000000000	Propionate and succinate producer; Lacks bft enterotoxin, lacks capsular polysaccharide LPS A; xylan and other plant polymers as C-source; proposed probiotic.
*Clostridium butyricum* GGCC_0151	Clostridiaceae	+				7-α-HSD, CGH, Taurine uptake	I	JABFCF000000000	Positive effects on IBS and IBD, but some reports on pathogenicity. Cluster I Clostridium used as probiotic. Induces IL-10 in experimental colitis model.
*Clostridium scindens* GGCC_0168	Lachnospiraceae					7-α-DH, 3-α-DH, 7-α-HSD, CGH, LCD	Clostr. G21	JABFCG000000000	Key for bile acid conversion via 7α-dehydratase activity and 3α-hydroxy bile acid-CoA-ester-3 dehydrogenase; Bacteriocin synthesis (Lactococcin 972 - like).
*Intestinimonas butyriciproducens* GGCC_0179	Ruminococcaceae	+				LCD	unassignedbut not IV	JABFHK000000000	Butyrate producer; linked to decreased susceptibility to gut inflammation; bacteriocin synthesis (Linocin M18-like).
*Eubacterium callanderi* GGCC_0197	Eubacteriaceae	+		+		7-α-HSD, CGH, SBS	XV	JABFAG000000000	Ameliorates experimental colitis and metabolite of microbe attenuates colonic inflammatory action with increase of mucosal integrity; butanol producer. Linocin M18-like bacteriocin synthesis and resistance operon.
*Extibacter sp.* GGCC_0201	Lachnospiraceae					7α/β -DH, LCD	Extibacter	JABFCH000000000	Key for bile acid conversion via 7-α-DH activity; lantipeptde synthesis (LanM plus precursor peptide).
*Akkermansia sp.* GGCC_0220	Akkermansiaceae		+	+	+	SBS	N.A.	JABFCI000000000	Based on ANI different species as A. municiphila ATCC BAA-835. Encodes lipopeptide for TLR2 dependent interaction with the innate immune system to control inflammation.
*Clostridium symbiosum* GGCC_0272	Lachnospiraceae	+				7-α-HSD, CGH, LCD, Taurine uptake	XIVa; Clostr. G24	JABFCJ000000000	Decrease negatively associated with severity of ulcerative colitis. Yersiniabactin synthesis operon.
*Bacteroides uniformis* GGCC_0301	Bacteroidaceae		+	+	+	CGH, 3-oxo-5-α, SBS	N.A.	JABFCK000000000	Positive effect on metabolic and immune dysfunction; no or low-level colitis induction compared to other Bacteroides species.
*Bitterella massiliensis* GGCC_0305	Lachnospiraceae	+				3-α-HSD, 3-β-HSD	XIVa	JABFCL000000000	Non-spore-forming (Durand et al, 2017); Key for bile acid conversion via 3α-hydroxy steroid dehydrogenase and 3β-hydroxy steroid dehydrogenase activities.
*Barnesiella sp.* GGCC_0306	Porphyromonadaceae S24-7		+	+		CGH. 3-oxo-5-α	N.A.	JABFCM000000000	Related strains reported to control vancomycin resistant Enterococcus faecium.

Genes involved in the deconjugation of bile salts and the conversion to secondary bile acids are indicated (+).

*CGH* choloylglycine hydrolase, *LCD* L-cantinine hydratase, *3-oxo-5α* 3-oxo-5-alpha-steroid-4-dehydrogenase, *7α-HSD* 7α-hydroxy steroid dehydrogenase, *3α-CHD* 3α-hydroxycholate dehydrogenase, *3α-DH* 3α-hydroxy bile acid-CoA-ester-3 dehydrogenase, *7α-DH* 7α-dehydratase, *7β-DH* 7β-dehydratase, *SBS* sodium-bile acid symporter system.

### GUT-108 successfully establishes in gnotobiotic mice models

**Fig. 3:**
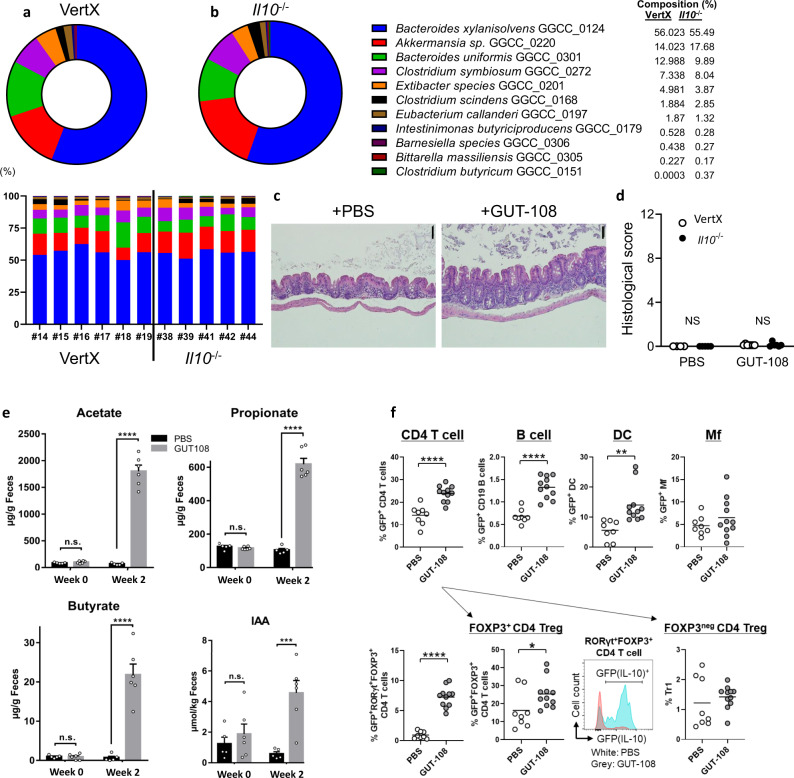
Effect on the gut microbiome community composition, colonic histology, and fecal metabolomics of gnotobiotic mice inoculated with GUT-108 or treated with PBS for 2 weeks. *Il10*^*+*^*/*^*eGFP*^ VertX reporter mice (**a**) or *Il10*^*−/−*^ mice (**b**) were inoculated via gavage with GUT-108 (2 × 10^+7^ cfu/strain), and after 2 weeks community composition of the gut microbiome was determined on cecal contents using qPCR with species-specific primers. The average community composition (circle diagram) as well as the individual community composition for six *Il10*^*+*^*/*^*eGFP*^ VertX reporter mice and five *Il10*^*−/−*^ mice are presented. The lower detection limit for qPCR was >0.0001%. **c** Representative distal colonic photomicrographs of H&E-stained tissue showing the lack of colonic inflammation two weeks after inoculation of gnotobiotic *Il10*^*−/−*^ mice with GUT-108 or PBS. The experiment was repeated independently with similar results for each animal in the GUT-108 (*N* = 11) or PBS (*N* = 8) group. The bar (upper right corner) in the pictures indicates 100 µm. **d** Histological scoring showing the lack of colonic inflammation 2 weeks after inoculation of gnotobiotic *Il10*^*+*^*/*^*eGFP*^ VertX reporter mice or *Il10*^*−/−*^ mice with GUT-108: combined score of cecum, proximal colon, and distal colon/rectum. Two-Way ANOVA with Sidak’s multiple comparison test. NS not significant. *N* = 5–6/group. **e** Metabolite analysis of fecal material from *Il10*^*−/−*^ mice inoculated with GUT-108 to confirm the successful restoration of bacterial synthesis of acetate, propionate, butyrate and IAA. Two-Way ANOVA with Sidak’s multiple comparison test. Bar indicates mean ± SE. ***P < 0.001, ****P < 0.0005, N.S. not significant. *N* = 8/group or *N* = 11/group, for PBS and GUT-108 treatment, respectively. **f** Colonization of gnotobiotic *Il10*^*+*^*/*^*eGFP*^ reporter mice with GUT-108 for 2 weeks induced IL-10-producing T and B cells, dendritic cells (DC) and macrophages (Mf) (top panel) and different types of IL-10^+^ regulatory T cells (lower panel^)^. GUT-108 refers to *Il10*^*+*^*/*^*eGFP*^ mice treated with GUT-108 (*N* = 11*)*; PBS refers to *Il10*^*+*^*/*^*eGFP*^ mice treated with PBS buffer (*N* = 8). Dots represent individual mouse results. Bars indicate mean. Mann–Whitney unpaired two-tailed *t*-test. Mean ± SE. *P < 0.05; **P < 0.01; ***P < 0.005; ****P < 0.001. *N* = 8 for PBS, *N* = 11 for GUT-108. Source data and calculated *P*-values are provided as a Source Data file.

GUT-108 was gavaged into two different GF models, *Il10*^*+/eGFP*^ VertX reporter and *Il10*^*−/−*^ mice, with engraftment in both *Il10*^*+*^*/*^*eGFP*^ VertX reporter (Fig. 3a) and *Il10*^*−/−*^ mice (Fig. 3b). Colonization levels of individual strains were determined using quantitative PCR. Colonization with GUT-108 did not induce inflammation as documented by representative distal colonic photomicrographs of H&E-stained tissue (Fig. 3c) and blinded histologic scores (Fig. 3d). Importantly, GUT-108 colonization promoted a healthy gut microbiome with key functionalities for SCFA, especially synthesis of acetate, butyrate and propionate, and IAA synthesis (Fig. 3e). Four GUT-108 strains, *Clostridium butyricum* GGCC_0151, *Intestinimonas butyriciproducens* GGCC_0179, *Bitterella massiliensis* GGCC_0305, and *Barnesiella* sp. GGCC_0306 were established at levels below 1% in both *Il10*^*+*^*/*^*eGFP*^ VertX reporter (Fig. 3a) and *Il10*^*−/−*^ mice (Fig. 3b), with *Clostridium butyricum* GGCC_0151 failing to get established in *Il10*^*+*^*/*^*eGFP*^ VertX reporter mice. However, due to redundancies in metabolic interdependencies and therapeutic functions, low luminal concentrations of these strains did not prevent restoring key metabolic phenotypes of a healthy gut microbiome. The ability of the GUT-108 consortium to stimulate regulatory (protective) IL-10-mediated immune responses was determined by comparing the type and levels of IL-10-producing immune cells in *Il10*^*+*^*/*^*eGFP*^ *VertX* reporter mice. Two weeks after being introduced, GUT-108 stimulated higher numbers of colonic LP IL-10-producing CD4^+^ T cells, B cells, and dendritic cells (DC) and increased numbers and percentages of regulatory T cells, including inducible Tregs (IL-10^+^ RoRγT^+^ FoxP3^+^ CD4^+^ cells and IL-10^+^ FoxP3^+^ CD4^+^ cells) (Fig. 3f). Although showing an upward trend, no statistical difference was observed for FoxP3^neg^ IL-10^+^ T cells (TR_1_).

### GUT-108 reverses gut microbiome dysbiosis, restores functionality

**Fig. 4:**
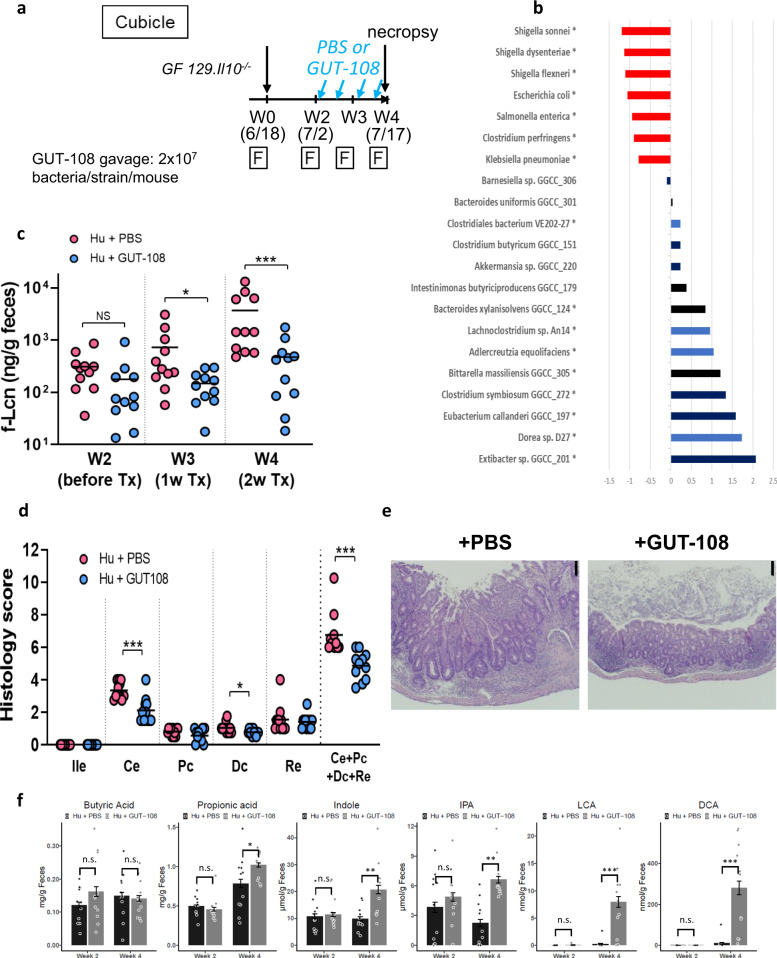
GUT-108 strains decreased colitis in Il10^*−/−*^ mice colonized with human fecal microbiota. **a** Schematic overview of experimental design. On day 1, mice were inoculated with a human stool previously verified to induce aggressive colitis in gnotobiotic *Il10*^*−/−*^ mice. Therapeutic application of GUT-108 or PBS control started after 2 weeks. After 4 weeks necropsies were performed, and tissue samples were collected for analysis. **b** Bacterial species whose abundance significantly changed in the gut microbiome of mice that received therapeutic application of GUT-108 compared to PBS control mice. Differences in relative species abundance between the two treatments is expressed on a Log10 scale. Strains with decreased (red) or increased (blue) relative abundance are indicated. GUT-108 strains are indicated in black. *P < 0.05 value using the two-sided Benjamini–Hochberg corrected Wilcoxon test. **c** Serial fecal lipocalin-2 levels were measured using ELISA after 2 (before start of GUT-108 or PBS therapy), 3 and 4 weeks. Dots indicate individual mice data. Mann–Whitney unpaired two-tailed *t*-test. Bars indicate Mean ± SE, *P < 0.05, ***P < 0.001, N.S. indicates not significant. *N* = 11/group. **d** Blinded histologic scoring assessed the level of inflammation for different parts of the intestine, including the distal ileum (Ile), cecum (Ce), proximal- (Pc) and distal- (Dc) colon, rectum (Re), and combined (Ce + Pc + Dc + Re). Dots indicate individual mice data. Mann–Whitney unpaired two-tailed *t*-test. Bars indicate Mean ± SE. Unpaired two-tailed *t*-test was performed on the data. *P < 0.05, ***P < 0.001, NS not significant. *N* = 11/group. **e** Representative distal colonic photomicrographs of H&E-stained tissue showing colonic tissues 2 weeks after starting therapeutic treatment of humanized *Il10*^*−/−*^ mice with GUT-108 or PBS. The experiment was repeated independently with similar results for each animal in the GUT-108 (*N* = 11) or PBS (*N* = 11) group. The bar in the picture indicates 100 µm; Hu + GUT-108 refers to humanized *Il10*^*−/−*^ mice treated with GUT-108; Hu + PBS refers to humanized *Il10*^*−/−*^ mice that received PBS as a placebo control. **f** Induction of protective fecal metabolites by therapeutic GUT-108 treatment beginning at 2 weeks after the induction of inflammation by human stool. Week 4 represents 2 weeks of therapeutic treatment. Hu+GUT-108 refers to humanized *Il10*^*−/−*^ mice treated with GUT-108; Hu + PBS refers to humanized *Il10*^*−/−*^ mice that received PBS buffer. DCA deoxycholic acid, LCA lithocholic acid, IPA indole-3-propionic acid. Two-way ANOVA and adjusted *P*-values were calculated by the multiple comparisons test. Bar indicates Mean ± SE. N.S. not significant; *: adjusted *P* < 0.05; **: adjusted *P* < 0.01; ***: adjusted *P* < 0.001. Source data and calculated *P*-values are provided as a Source Data file.

Therapeutic application of GUT-108 was tested in a newly developed experimental colitis model using GF *Il10*^*−/−*^ mice (129SvEv background) humanized with a fecal transplant from a healthy human donor (Fig. 4a). Experimental colitis was induced by inoculating the GF *Il10*^*−/−*^ mice with stool from the healthy donor that was previously verified to induce aggressive colitis in ex-GF *Il10*^*−/−*^ mice^24^. Over time the normal microbiota from a healthy donor are altered in an inflammatory environment^21,25,26^. We documented in Fig. 3c and Fig. 3d that gnotobiotic *Il10*^*−/−*^ mice selectively colonized with the GUT-108 strains did not develop colitis. The difference in outcomes of colitis in *Il10*^*−/−*^ mice colonized with the complex microbiota of the healthy human stool versus protection with GUT-108 is likely because GUT-108 contains only protective, commensal bacteria, in contrast to the presence of pathobiont strains present at low levels in healthy human stool, but which expand in a pro-inflammatory environment. Therapeutic application of GUT-108 beginning 2 weeks after human fecal colonization changed the community composition (Supplementary Fig. 4) and reduced the expansion of opportunistic pathogenic bacteria belonging to the Enterobacteriaceae family that occurred in mice receiving PBS (Supplementary Table 3). Metagenome analysis showed that treatment with GUT-108 resulted in engraftment of all GUT-108 strains except *Clostridium scindens* GGCC_0168, restoring a gut microbiome that promoted the abundance of beneficial resident *Clostridium* (Clusters IV and XIVa) strains not part of the GUT-108 consortium. Compared to PBS therapy and consistent with the GUT-103 therapeutic results, GUT-108 decreased by 90% the abundance of *Enterobacteriaceae*, including *Klebsiella pneumoniae, Salmonella enterica, Escherichia coli*, and *Shigella* species, from 5% to below 0.5% (Fig. 4b).

The progression of colitis in humanized *Il10*^*−/−*^ mice was evaluated by serial fecal lipocalin-2 (Fig. 4c) after human fecal gavage. Two weeks after human fecal inoculation, elevated levels of fecal lipocalin-2 were observed (compared to basal lipocalin-2 levels of ~3 ng/g feces material), indicating the onset of moderate to severe colitis. Therapeutic application of GUT-108 at 2 weeks after human fecal transplantation decreased inflammation compared to the PBS treatment as measured by fecal lipocalin-2 (Fig. 4c) and blinded histologic scoring (Fig. 4d). GUT-108 decreased histologic inflammation in the cecum and distal colon, as well as the summed colonic score, which we used as an overall inflammatory index^20,21,27^. Representative photomicrographs of the distal colon demonstrated decreased inflammation following GUT-108 therapy with reduced mononuclear cell infiltration in the LP and submucosa, mucosal thickening and crypt hyperplasia (Fig. 4e). Metabolite analysis confirmed that the therapeutic application of GUT-108 restored a physiologically healthy gut microbiome that produced increased levels of anti-inflammatory bacterial metabolites associated with mucosal homeostasis, including the SCFA propionate and synthesis of indole and IPA (Fig. 4f). Likewise, therapeutic application of GUT-108 significantly increased levels of the secondary bile acids LCA and DCA (Fig. 4f), a key objective in the rational design of GUT-108. In contrast to propionate, treatment did not change butyrate levels, consistent with the findings of the selectively colonized gnotobiotic mouse models, where stool butyrate levels were 30-fold lower than those for propionate (Fig. 3e).

### Therapeutic application of GUT-108 reduces inflammatory cytokines

**Fig. 5:**
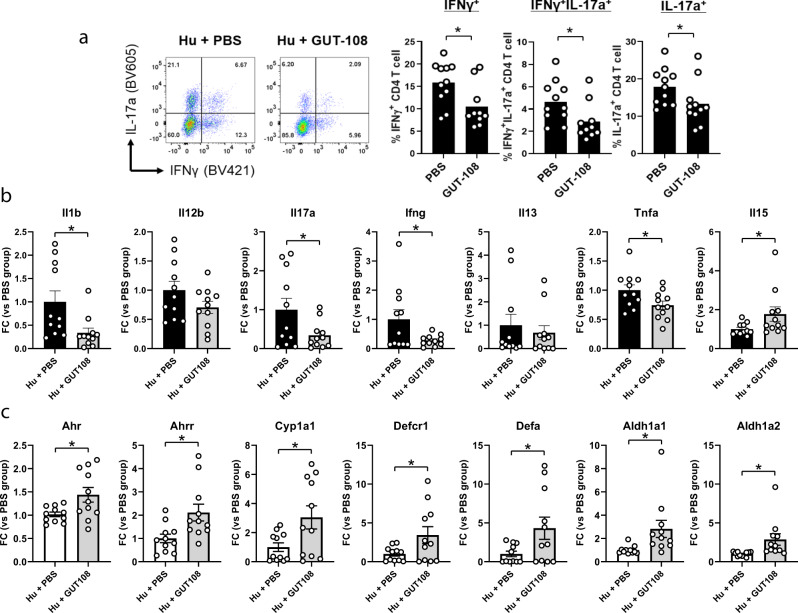
Effects of treatment with GUT-108 on the levels of inflammatory biomarkers. **a** Colonic lamina propria effector CD4 + T cells, including Th1 and Th17 cells that produce IFNγ and/or IL-17α. The mRNA expression levels of **b** inflammatory cytokines and **c** functions important for mucosal homeostasis in the distal colon. Expression levels were determined by RT-Q-PCR and presented as fold change (FC) of expression after therapeutic application of GUT-108 compared to expression after PBS treatment. Hu + GUT-108 refers to humanized *Il10*^*−/−*^ mice treated with GUT-108 2 weeks after fecal colonization; Hu+PBS refers to humanized *Il10*^*−/−*^ mice that received PBS as a placebo control. *AhR* aryl hydrocarbon receptor gene, *AhrR* aryl hydrocarbon receptor repressor gene, *Cyp1A1* Cytochrome P450 Family 1 Subfamily A Member 1gene, *DefCR1* and *DefA* genes defensins, *Aldh1A1* and *Aldh1A2* genes aldehyde dehydrogenases. Bar indicates Mean ± SE. Two-way ANOVA and adjusted *P*-value were calculated by the multiple comparisons test. *: adjusted *P* < 0.05. *N* = 11/group. Source data and calculated *P*-values are provided as a Source Data file.

Reduced severity of inflammation and improved mucosal immune homeostasis after therapeutic application of GUT-108 was characterized by decreased levels of colonic LP effector CD4^+^ T cells, including Th1 and Th17 cells that produce the inflammatory cytokines IFNγ and IL-17α, respectively, or both IFNγ and IL-17α (Fig. 5a); reduced distal colonic expression levels of innate and Th1 and Th17 pathway inflammatory cytokines, including IL-1β, IL-12p40, IL-13, IL-17α, IFNγ, and TNFα (Fig. 5b), and increased colonic expression levels of receptors and pathways implicated in mucosal healing (Fig. 5c, Supplementary Fig. 5).

Several protective pathway components that are decreased in IBD were upregulated 2 weeks after application of GUT-108, further supporting its therapeutic role and providing mechanisms for protection (Fig. 5c). Expression of the Ahr pathway genes, including the aryl hydrocarbon receptor (Ahr), the aryl hydrocarbon receptor repressor (AhrR), and the Cytochrome P450 Family 1 Subfamily A Member 1 (Cyp1A1) gene^28^, were significantly upregulated. Protective pathways with increased gene expression levels included the genes for the defensins DefCR1 and DefA, whose altered production is suggested to be integrally involved in IBD pathogenesis^29^, and the genes for aldehyde dehydrogenases Aldh1A1 and Aldh1A2, whose expression is reduced in colonic macrophage and DC subsets of patients with ulcerative colitis regardless of inflammation^30^ (Fig. 5c). Together these results confirm, by multiple readouts, that administration of GUT-108 treats established colitis in humanized mice, out-competes pathobionts, inhibits effector immune responses, and restores homeostatic metabolic and protective immune responses.

## Discussion

Resident bacterial strains of the GUT-103 and GUT-108 consortia, designed to interdependently restore normal function to the inflamed colon, rapidly colonized gnotobiotic mice as well as ex-germ-free humanized mice that had developed a pro-inflammatory gut microbiome. These GUT-103 and 108-colonized mice exhibited the desired functional properties consistent with the rational design of the two consortia.

The two separate rationally designed consortia of intestinal bacterial strains with redundant functionalities that were previously found to be under-represented in the gut microbiome of IBD patients with active disease^3^ were shown to promote homeostatic immune functions and bacterial metabolism and prevent onset or progression of intestinal inflammation. We believe that this approach has the potential to maintain long term remission in a physiologic and safe manner, which should be tested in a Phase 1 clinical trial.

Testing GUT-103 against EER-induced colitis in gnotobiotic *Il10*^*−/−*^ mice is clinically relevant to human Crohn’s disease because adherent-invasive *Escherichia coli* and *Ruminococcus gnavus* are increased in active Crohn’s disease^3,31,32^ and the adherent-invasive *E. coli* strain used was isolated from the ileum of a Crohn’s disease patient^33^. GUT-108 showed therapeutic efficacy to reverse established colitis in *Il10*^*−/−*^ humanized mice by intervening 2 weeks after onset of moderate-severe intestinal inflammation, supporting a potential role for GUT-108 to treat active IBD. Moreover, the *Il10*^*−/−*^ model of chronic pathobiont-driven T-cell-mediated chronic inflammation is more predictive of therapeutic responses in IBD patients than acute epithelial injury models such as dextran sodium sulfate^2^.

GUT-103 and GUT-108 were designed based on human data showing that protective functions provided by commensal bacteria are under-represented in the gut microbiome of IBD patients. These functions include the synthesis of SCFAs, indole and its derivatives, bile acid deconjugation and conversion, and competition for the critical nutrient iron and the synthesis of antagonistic molecules to control opportunistic pathogens^3,34^. Several animal studies have highlighted the role for SCFAs, especially propionate and butyrate, in regulatory T cell recruitment and function^8,12,13,34–36^. The recruitment in the colon and extrathymic conditioning of regulatory T cell response by SCFA make these molecules an important link in the crosstalk between the gut microbiome and the immune system. Therefore, commensal bacteria identified to produce propionate and butyrate were selected in the rational design of GUT-103 (Table 1) and GUT-108 (Table 2).

Several studies have shown the role of indole, a metabolite produced from tryptophan, and its metabolites in reducing attachment of pathogenic *E. coli* to epithelial cells^37^, strengthening the mucosal barrier and mucin stimulating production^38^. Therefore, commensal bacteria identified to produce indole and its derivatives were selected in the rational design of GUT-103 (Table 1) and GUT-108 (Table 2).

An inflammatory gut microbiota can result in the inefficient microbial conversion of bile salts into their primary and secondary bile acids^3^. IBD patients with an unbalanced gut microbiome due to inflammation have lower fecal and circulating concentrations of secondary bile acids and higher conjugated fecal bile acid concentrations than do healthy subjects^39^. Thus, activities essential for the conversion of primary bile acids, specifically the conversion of CA and TCDCA acid via a multistep process that includes 7-alpha-dehydroxylation by 7-alpha dehydratase (7-α-DH) or 7-alpha-hydroxy steroid dehydrogenase (7-α-HSD) activity^15^, are included in the strain selection for GUT-103 (Table 1) and GUT-108 (Table 2).

Competition for iron helps drive the competitiveness and establishment of microorganisms^40^. Therefore, GUT-103 (Table 1) and GUT-108 (Table 2) include several strains that synthesize one or more siderophores under iron-limiting conditions. Ideally, these siderophores are insensitive to inhibition by Lipocalin-2, a peptide that inhibits specific siderophores and their uptake, and is a major colonic defense system triggered by bacterial infections. Lipocalin-2 levels were increased after induction of inflammation in *Il10*^*−/−*^ mice colonized with the EER consortium (Fig. 1c) or human fecal microbiota (Fig. 4c).

Bacteriocins, of which lantibiotics are considered a specific class, have shown great promise as new antibiotics for therapeutic application, as reviewed by Field et al.^41^. Thus, bacteriocin synthesis was included as a key functionality in strain selection as part the rational design process of GUT-103 (Table 1) and GUT-108 (Table 2).

The optimized 11-strain GUT-108 consortium was rationally designed to build on the proof-of-concept results obtained with GUT-103. GUT-108 went beyond members of the *Clostridium* clusters IV and XIVa strains, including *Bacteroides* and *Akkermansia* species. Furthermore, based on their genome analysis, strains with undesirable properties including the presence of transferable antibiotic resistances or putative virulence factors were excluded. We also omitted species that are fastidiously anaerobic, such as *Faecalibacterium prausnitzii*, from the GUT-108 consortium. Compared to GUT-103, GUT-108 strains provide additional redundancy for the synthesis of the protective secondary bile acids LCA and DCA, plus multiple mechanisms to compete with opportunistic pathogenic Enterobacteriaceae including synthesis of the siderophore yersiniabactin, and lantibiotics. In the inflammatory gut environment of humanized *Il10*^*−/−*^ mice, beneficial Lachnospiraceae and Ruminococcaceae family members are decreased while opportunistic Enterobacteriaceae are increased^42^, as reported for Crohn’s disease patients^32,43^. Therapeutic application of GUT-108 reduced levels of colitogenic Enterobacteriaceae and increased beneficial resident *Clostridium* (Clusters IV and XIVa) species, especially Lachnospiraceae including *Dorea* species and *Lachnoclostridium* species that are not GUT-108 constituents (Fig. 4b). This altered community composition increased cecal luminal propionate concentrations, but not butyrate levels (Fig. 4f). Previous studies demonstrated that butyrate levels are not necessarily an indicator of a healthy gut microbiome, as butyrate synthesis from fermentation of amino acids such as lysine can contribute to inflammation under conditions associated with mucosal permeability^44^.

GUT-108 increased expression of *Gpr41* and showed an upward trend for *Gpr43* in *Il10*^*−/−*^ mice humanized with a fecal transplant (Supplementary Fig. 5). As previously reported, SCFA produced by gut bacteria stimulate Tregs^8^ with propionate’s effect mediated through GPR43^36^. SCFA also mediate the function of GPR41, a key regulator that controls host energy balance^45^.

With its functional redundancy and metabolically interdependent auxotrophies, GUT-108 is designed to engraft and perform under a wide range of conditions. When applied to *Il10*^*−/−*^ mice humanized with a fecal transplant, all GUT-108 strains except *Clostridium scindens* GGCC_0168 were established for at least 2 weeks. *Clostridium scindens* has been previously described as one of the essential strains necessary to convert primary bile acids into LCA and DCA. However, despite the absence of this strain, the established functional multi-strain network produced secondary bile acids, with *Extibacter* sp. GGCC_0201 providing the 7α-dehydratase activity required to convert CA and CDCA into the therapeutic secondary bile acids DCA and LCA, respectively. Normalizing the intestinal bile acid profile can restore intestinal epithelial stem cell function^46^, and increase colonic RORγ^+^ Treg cell counts that ameliorate host susceptibility to colitis^47^, while LCA stimulates Treg differentiation and inhibits Th17 cells^48^ consistent with GUT-108’s ability to restore secondary bile acid metabolism (Fig. 4f) and activate inducible IL-10^+^ RORγ FoxP3^+^ CD4^+^ Treg cells (Fig. 3f). GUT-108 stimulated regulatory (protective) immunity by increasing numbers of colonic LP IL-10-producing CD4^+^ T cells, B cells and DC and numbers and percentages of regulatory T cells, including inducible Tregs (IL-10^+^ RORγT^+^ FoxP3^+^ CD4^+^ cells) and IL-10^+^ Tregs (Fig. 3f). We further documented the anti-inflammatory effects of therapeutic GUT-108 in *Il10*^*−/−*^ mice humanized with a fecal transplant by demonstrating that GUT-108 decreased IFN-ɣ^+^, IL-17α^+^, and IFN-ɣ^+^ IL-17α^+^ synthesizing colonic LP CD4^+^ TH_1_ and TH_17_ cells (Fig. 5a) and reduced expression levels of innate and Th1 and Th17 pathway cytokines, including IL-1β, IL-12p40, IL-13, IL-17α, IFNγ, and TNFα (Fig. 5b). Interestingly, GUT-108 treatment increased expression of IL-15 mRNA, a homeostatic cytokine that controls T cell inflammatory responses. Exogenous IL-15 treatment decreases IL-17α expression by Th17 cells in vitro through STAT5 enrichment at the *IL-17* locus^49^, consistent with the ability of GUT-108 therapy to increase IL-15 gene expression and decrease IL-17α mRNA expression in colonic LP cells (Fig. 5b).

Increased intestinal bacterial metabolism of tryptophan, especially indole and its derivatives IAA and IPA, activates the *Ahr* pathway. AHR acts as a sensor of the microbiota community and, through its established role of modulating immune functions, maintains host-microbe homeostasis^50^. IPA is also a pregnane X receptor (PXR) agonist mediating its responses through TLR4^51^. GUT-108 therapy increased both IAA and IPA levels in stool (Fig. 4f) and colonic *Ahr* gene expression (Fig. 5c). AHR is a critical mediator of anti-inflammatory responses to infection by bacterial pathogens and of the differentiation and function of immune cells including T cells, innate lymphoid cells, macrophages and DC^28^. AHR promotes the expression of the anti-inflammatory cytokine IL-10 and inhibits macrophage apoptosis, decreases the expression of inflammatory cytokines (IL-6 and TNF-α) and inhibits activation of NF-κB. Therefore, the *Ahr* pathway is critical to protect from excessive inflammatory cytokine expression and septic shock. In addition, *Ahr* pathway activation protects the mucosa during inflammation^52^.

Efficacy of GUT-108 in *Il10*^*−/−*^ mice is mediated by IL-10-independent mechanisms. Further insights into these possible protective mechanisms include increased expression of metabolite sensors and mediators (*Gpr41, Gpr43, Fxr, Pxr, Pparg, Fgf15, Fgf21*) and pathways mediating differentiation of immune cells including Treg and Breg cells (*cMaf, Il5, April, Aid, Bcl6*) (Supplementary Fig. 5). For example, intestinal epithelial FGF15 is activated by bile acids serving as ligands for the nuclear receptor farnesoid X receptor (FXR). FXR/FGF15 signaling regulates bile acid homeostasis and protects against experimental colitis^53^. APRIL impacts immune regulatory T cells by stimulating their proliferation and survival, and directly contributing to their immune suppression^54^. Bcl6 induces IL-10 and follicular T helper cells and regulates the balance of innate lymphoid cells subsets^55^. Decreased expression of *Nos2* (Supplementary Fig. 5), a part of the Ace2 -Nos2-IFNɣ biosynthesis gene cascade^56^, could lead to lower levels of ACE2, a key regulator to control intestinal inflammation induced by epithelial damage^57^. Certain viruses, including the coronaviruses SARS-CoV-1 and SARS-CoV-2, use the ACE2 protein for infecting respiratory and intestinal epithelial^58^. Therefore, chronic gut inflammation, as seen in type-2 diabetes and obesity^59^ might trigger elevated gut epithelial ACE2 levels, and therefore patients suffering from these conditions could be more sensitive for Coronavirus infection and at higher risk for complications, highlighting the importance of therapeutically targeting the pro-inflammatory gut microbiome as the underlying cause of chronic inflammation^60^.

In addition to analyzing fecal material, we measured cecal microbiota and in some cases cecal luminal metabolites to represent bacterial communities and function within the cecum/colon, since the cecum is one of the sites of most active inflammation (Fig. 4d). Previous rodent studies have shown broadly similar microbial patterns in cecal luminal and fecal samples^61^. Furthermore, we demonstrate similar cecal (Fig. 2) and fecal (Fig. 4f) secondary bile acid (LCA and DCA) responses to GUT-103 and GUT-108, respectively.

Although no animal model completely replicates all clinical features of human Crohn’s disease or ulcerative colitis, we believe that our use of a human fecal transplant in germ-free mice to initiate chronic TH_1_/TH_17_-mediated colitis and our treatment protocol of administering GUT-103 and 108 to mice with established mild to moderate inflammation replicates IBD as closely as feasible for preclinical studies. Proof of efficacy in human IBD will have to await a clinical trial.

GUT-103 and GUT-108 combine multiple modes of action to treat the upstream causes of inflammation by correcting the abnormal microbiome environment, activating various IL-10 synthesizing immune cells, lowering inflammatory responses, and restoring bacterial metabolic profiles to levels found in stool samples of healthy individuals. These overlapping protective mechanisms are predicted to maintain long term remission of IBD in a physiologic and safe manner, in contrast to most biologicals, which block downstream immune effector responses by neutralizing a single cytokine or molecule and induce immunosuppression that can be associated with increased infection and neoplasms. These integrated protective mechanisms make GUT-108 a promising novel therapy to treat a range of conditions whose pathogenesis is characterized by dysbiosis-mediated chronic intestinal inflammation and increased mucosal permeability. Besides IBD this could include graft versus host disease, hepatic encephalopathy, alcoholic liver disease, atherosclerosis, hypertension, obesity, metabolic syndrome, and type-2 diabetes mellitus.

## Methods

### Ethics statement

All animal experiments were performed in compliance with all relevant ethical regulations for animal testing and research as described in the National Institutes of Health Guide for the Care and Use of Laboratory Animals. The study received ethical approval by the University of North Carolina—Chapel Hill Institutional Animal Care and Use Committee (18-266.0-B).

The human fecal sample for transplant to mice was collected in compliance with all relevant ethical regulations for work with human participants, and with informed consent from the participant under the approved University of North Carolina Institutional Review Board protocol #17-1528.

### Microbiology techniques

All bacterial strains were grown on LYH-BHI medium (Brain–heart infusion medium supplemented with 0.5% yeast extract (Difco)), except *Bitterella massiliensis* GGCC_0305 and *Barnesiella* sp. GGCC_0306, which were grown on Peptone Yeast Glucose broth (Anaerobe Systems). Strains were cultivated at 37 °C under strict anaerobic conditions (H_2_ (5%), CO_2_ (10%)-, and N_2_ (85%) v/v/v). After growth, cultures were concentrated at 10^+9^ cfu/ml in phosphate buffered saline (PBS). Cultures were tested for contamination with aerobic bacteria by streaking on LB medium, followed by incubation at 37 °C under aerobic conditions. For quality control, a culture was considered pure if no aerobic growth was observed and the sequenced 16 S rRNA gene of each culture matched the anticipated composition.

### DNA sequencing

Bacterial genome sequencing libraries were generated using the ThruPLEX DNA-seq Kit (Rubicon Genomics). Individual strain libraries were combined in equimolar proportions in one pool and sequenced by 125 bp pair end read sequencing on the Illumina HiSeq2500. Onboard image processing and base calling were performed. The sequence data quality score (Q score) was used as a quality control metric with the specification that ≥80% of bases must have a Q score of ≥30. After trimming, sequencing reads were assembled with SPAdes (version 3.13.0) using default parameters^62^ and annotated using Prokka (version 1.14-dev)^63^ and the RAST server^64–66^. Genome sequences for GUT-108 strains were submitted to GenBank (Table 2).

DNA from fecal and cecal material was extracted with AllPrep PowerViral DNA/RNA Kit (Qiagen). Metagenome sequence libraries were constructed using the KAPA Hyper DNA library prep. DNA libraries were multiplexed and loaded on an Illumina HiSeq4000 instrument per manufacturer’s instructions. Metagenome sequencing was performed using a 2 × 150 paired-end configuration; image analysis and base calling were conducted by the HiSeq Control Software (v2.0.12) on the HiSeq instrument. Sequencing reads were trimmed, and mouse reads were filtered out using Trimmomatic (version v0.39)^67^. Species abundance was determined directly on the metagenome sequencing reads with Kaiju (version v1.7.2)^68^. The raw sequencing reads from the fecal samples have been deposited with links to BioProject accession number PRJNA703330 in the NCBI BioProject database (https://www.ncbi.nlm.nih.gov/bioproject/).

### Multi-genome analysis and modeling

Genome sequences for GUT-108 strains or reference strains, downloaded from GenBank files (National Center for Biotechnology Information, NCBI) or Pathosystems Resource Integration Center (PATRIC)^17^ were consistently annotated using Prokka (version 1.14-dev), the RAST server, antiSMASH (version 5.1.1)^69^, and Blast against custom-built protein databases. Models were reconstructed, gapfilled, and Flux Balance Analysis^70^ (FBA) was run using the KBase fbatools module v2.0, which is currently deployed in beta mode in the KBase narrative interface (https://narrative.kbase.us/). Reconstruction was performed using the “Build metabolic model” app; gapfilling was performed using the “Gapfill Metabolic Model” app; all flux balance analysis was performed using the “Run Flux Balance Analysis” app; community models were assembled using the “Merge Metabolic Models into Community Model” app; and community flux balance analysis was performed using the “Run Flux Balance Analysis” app. As a last step, all models were manually curated and refined prior to final analysis. Auxotrophies were determined by reviewing the Pathway table to determine if the compounds were created via a reaction or combination of reactions. ‘+’ indicates the compound is produced. “AA” indicates the compound is not produced. Average nucleotide identity was determined via pyani (version 0.2.7.dev)^71^. The results are presented in Supplementary Data files 1to 6.

### Quantitative (RT)-PCR of tissue RNA

For RNA stabilization of colon tissue, RNAlater (Qiagen) was used. The total RNA extractions were performed with RNeasy Plus Mini kit (Qiagen, MD, USA). cDNA was created with the SensiFAST cDNA synthesis kit (Bioline, TN, USA) by PCR (25 °C, 10 min; 42 °C, 15 min; 85 °C, 5 min). Quantitative (RT)-PCR was performed with QuantStudio3 (Thermo Fisher Scientific, PA, USA) using SYBR No-ROX reagents (Bioline, TN, USA) with the following PCR setting: 95 °C, 2 min; 95 °C, 5 sec; 40 cycles of (60 °C, 10 sec; 72 °C, 20 sec); melting curve analysis: 95 °C, 15 sec; 60 °C, 15 sec; 95 °C, 15 sec. The data were created by comparative Ct method (2^-ΔΔCt^). Each cDNA sample was analyzed in duplicate for quantitative assessment of RNA amplification. Melting curve analysis confirmed the presence of single products with expected melting temperatures. An overview of the primers used is provided in Supplementary Data 7.

### Quantitative (RT)-PCR of bacterial DNA

Q-PCR using strain-specific primers against the single copy *RpoB* gene were used to quantify the composition of the GUT-103 and GUT-108 consortia after gavage in gnotobiotic mice. *RpoB* primers were validated to ensure there was no significant cross-reactivity between their target bacterium and other bacteria that were present in the consortia (Supplementary Fig. 6). The strain-specific *RpoB* primers are provided in Supplementary Data 7. The genomic DNA was extracted from fecal or culture media using AllPrep PowerViral DNA/RNA Kit (Qiagen). Q-PCR were performed with QuantStudio3 (Thermo Fisher Scientific, PA, USA) using SYBR No-ROX reagents (Bioline, TN, USA) and 10 ng of genomic DNA per strain with the following PCR settings: 95 °C, 3 min; 40 cycles of (95 °C, 5 sec; 60 °C, 20 sec); melting curve analysis: 95 °C, 15 sec; 60 °C, 15 sec; 95 °C, 15 sec. The data were created by comparative Ct method (2^-Ct^). Melting curve analysis confirmed the presence of single products with expected melting temperatures.

### Targeted metabolomics

Targeted metabolomics were conducted to determine concentration levels of bile acids, short-chain fatty acids (SCFAs), and tryptophan/indole metabolites in mouse feces and/or cecal contents. Organic solvents and reagents of LC/MS-grade (Optima^TM^), including methanol (MeOH), isopropanol (IPA), acetonitrile (ACN), water, and formic acid (FA), were purchased from Fisher Scientific (Waltham, WA, USA). Reagents of analytical grade, including 1-propanol (PrOH), pyridine (Py), propyl chloroformate (PCF), alongside semiconductor-grade sodium hydroxide (NaOH), and anhydrous sodium sulfate (Na_2_SO_4_), were purchased from Sigma–Aldrich (St. Louis, MO, USA).

For SCFA analysis, authentic reference chemical standards of acetic acid (AA), propionic acid (PA), and butyric acid (BA) were purchased from Sigma–Aldrich (St. Louis, MO, USA); stable isotope-labelling (SIL) d4-AA, d2-PA, and d2-BA were procured from Fisher Scientific (Waltham, MA, USA). Fecal SCFA extraction and measurement methods were performed as previously described with minor modifications^72^. In brief, ~40 mg sample aliquots were extracted on a TissueLyzer (Qiagen, Hilden, Germany) into 0.005 M NaOH aqueous solution spiked with 50 μg/mL of d4-AA, 10 μg/mL of d2-PA, and 10 μg/mL of d2-BA, followed by centrifugation at 13,200 rpm for 20 min. The supernatant was transferred to glass tubes and derivatized by sequential addition of water, PrOH:Py (3:2, *v/v*), and PCF. Upon capping, brief vortexing and two-min sonication was performed. The propyl derivatives of SCFAs were extracted twice with hexane, combined, and transferred to Na_2_SO_4_-containing vials for GC-MS quantitation. Instrumental analysis was performed on an Agilent 7820 A gas chromatography system coupling to a 5977B mass spectrometric detector (MSD) (Santa Clara, CA, USA), where 1 µL of derivatives was injected under 1:10 split mode for electron ionization and separated by a DB-5ms column (30 × 0.25 × 0.25 mm) (Santa Clara, CA, USA) with 1 mL/min helium flowing through. The GC oven temperature program was set as follows: initial oven 175 temperature held at 50 °C for 2 min, ramped to 70 °C by 10 °C/min, to 85 °C by 3 °C/min, 176 to 110 °C by 5 °C/min, to 290 °C by 30 °C/min, and finally held at 290 °C for 8 min; the MSD was operated at full scan mode with scan range of *m/z* 30–600. The relative standard deviations of intra- and interday precision of analysis were below 10%. Lower limits of quantitation (LLOQ) were determined to be 1 ng for AA (on column), 1 ng for PA, and 0.1 ng for BA.

For bile acid analysis, authentic reference standards (in salt forms) of 23 bile acids, including dihydroxycholestanoic acid (DHCA), glycoursodeoxycholic acid (GUDCA), α-hyocholic acid (HCA), taurochenodeoxycholic acid (TCDCA), cholic acid (CA), taurodeoxycholic acid (TDCA), glycodeoxycholic acid (GDCA), chenodeoxycholic acid (CDCA), deoxycholic acid (DCA), lithocholic acid (LCA), ursodeoxycholic acid (UDCA), tauroursodeoxycholic acid (TUDCA), murideoxycholic acid (MDCA), glycocholic acid (GCA), glycohyocholic acid (GHCA), glycochenodeoxycholic acid (GCDCA), tauro-ω-muricholic acid (TωMCA), tauro-α-muricholic acid (TαMCA), tauro-β-muricholic acid (TβMCA), taurocholic acid (TCA), α-muricholic acid (αMCA), β-muricholic acid (βMCA), ω-muricholic acid (ωMCA) were purchased from Cayman Chemical (Ann Arbor, MI, USA) and Steraloids Inc. (Newport, R.I., USA). Synthetic SIL internal standards, including d4-GCA, d6-GDCA and GDHCA, d4-GLCA, d4-GUDCA, d4-TβMCA, d4-CA, d4-TUDCA, d9-TCDCA, d6-TDCA, d4-UDCA, d9-CDCA, d6-DCA, and d4-LCA were purchased from Cambridge Isotope Laboratories, Inc. (Tewksbury, MA, USA). Bile acids in feces and/or cecal content were determined using LC-MS/MS based on previously published procedures with slight modifications^73,74^. Briefly, ~10 mg cecal contents were extracted with ACN and MeOH. Twenty microliter of 1 µM internal standard mixture of bile acids was spiked in prior to the extraction. The samples were homogenized on a TissueLyzer (Qiagen, Hilden, Germany) followed by centrifugation at 16,000 rpm for 10 min. Both supernatants were combined, centrifuged at 16,000 rpm for 10 min again, SpeedVac dried, reconstituted with MeOH, and diluted to 1.2 mL with 0.1% FA in water. The mixture extracts were passed through Biotage ISOLUTE^®^ C18 cartridges (100 mg/1 mL) (Uppsala, Sweden) which had been washed with MeOH and conditioned with MeOH:water (with 0.1% FA) (1:5, *v/v*) prior to analysis. Upon completion of sample loading, the cartridges were washed with MeOH:water (with 0.1% FA) and subject to vacuum drying. The analytes were eluted afterwards with MeOH, SpeedVac dried at 8 °C, and resuspended in 50% MeOH upon analysis. The instrumental analysis was conducted on a Vanquish UHPLC system coupling to a TSQ Quantis triple quadrupole mass spectrometer (Thermo Scientific, Waltham, MA, USA) interfacing with an electrospray ionization (ESI) source operated in negative ion mode. Chromatographic separation was carried out on an ACQUITY UPLC HSS T3 (2.1 × 100 mm, 1.8 µm) column (Milford, MA, USA) maintained at 60 °C. The mobile phases consisted of 0.01% FA in water (A) and 0.01% FA in ACN (B). The bile acids are eluted at a flow rate of 400 µL/min with a gradient elution of mobile phase starting at 25% B, held for 2 min; increased to 40% B in 13 min; increased to 98% B at 20 min, held for 2 min; decreased to 25% B at 22.5 min, and held for 3.5 min, for a total run of 26 min. The source parameters were: spray voltage, 3500 V; sheath gas, 50 arbitrary unit; aux gas, 25 arbitrary unit; sweep gas, 6 arbitrary unit; ion transfer tube temperature, 325 °C; vaporizer temperature, 350 °C. Quantification of bile acids was based on an isotope dilution method using the responses of d4-GCA (for GCA and GHCA), d6-GDCA (for GDCA and GDHCA), d4-GLCA (for GLCA), d4-GUDCA (for GCDCA, GHDCA, and GUDCA), d4-TβMCA (for TαMCA, TβMCA, TωMCA), d4-CA (for THCA, TCA, αMCA, βMCA, ωMCA, CA, and HCA), d4-TUDCA (for TUDCA), d9-TCDCA (for TCDCA), d6-TDCA (for TDCA and TDHCA), d4-UDCA (for UDCA), d9-CDCA (for CDCA and MDCA), d6-DCA (for DCA and DHCA), and d4-LCA (for LCA). Calibration curves with at least ten points spanning concentration ranges of 0.1–2000 nM were employed. The data were corrected for differences in the amount of stool analyzed. Regression coefficients R^2^ > 0.99 for all calibration curves. LODs for all bile acids were determined as below 0.5 nM.

For analysis of tryptophan and indole metabolites, reference chemical standards of tryptophan (Trp), kynurenine (Kyn), indole, indole-3-acetate (IAA), and indole-3-propionate (IPA), were purchased from Sigma–Aldrich (St. Louis, MO, USA). SIL internal standards for each compound, except d6-Kyn from Cambridge Isotopes Laboratories (Tewksbury, MA, USA), were obtained from CDN Isotopes (Pointe-Claire, Quebec, Canada). Tryptophan and indole compounds were determined using a LC-MS assay. In brief, ~20 mg feces and/or cecal content aliquots were extracted on a TissueLyzer (Qiagen, Hilden, Germany) with ice-cold MeOH:water solution (1:1, *v/v*) spiked with 500 nM internal standard mixtures. Upon centrifugation at 15,000 rpm for 10 min, the supernatant layer was collected, dried in a Labconco CentriVap vacuum concentrator (Kansas, MO, USA), and resuspended in ACN:water (2:98, *v/v*) solution upon instrumental analysis. A Thermo Vanquish UHPLC system coupled to a Q Exactive orbitrap mass spectrometer (Waltham, MA, USA) was used; injected analytes underwent 15-min chromatographic separation on an ACQUITY UPLC HSS T3 column (Waters, Milford, MA) and positive electrospray ionization (ESI), and ions were detected under parallel reaction monitoring (PRM) mode. Internal standard quantitation was performed disparately to determine the concentration levels of the five metabolites, using calibration curves with at least five points and a fitted linearity regression of R^2^ > 0.99. LLOQ were determined as 50 femtomole (fM) on column for Kyn and Trp, 100 fM for IAA and IPA, and 0.5 picomole (pmol) for indole. Recovery rates from sample extraction ranged from 80.5 to 100.7% for the five compounds, and no observable matrix effects were found throughout the analytical procedures.

### Mice

8-12 week-age GF 129SvEv background IL-10-deficient mice (*Il10*^*−/−*^) were obtained from University of North Carolina National Gnotobiotic Rodent Resource Center^20^. *Il10*-eGFP-reporter (*Il10*^+/eGFP^) mice on a C57BL/6 J background mice were originally provided by Dr. C. L. Karp (Global Health, Bill & Melinda Gates Foundation, USA) and raised in the National Gnotobiotic Rodent Resource Center. Germ-free and gnotobiotic mice were maintained in positive-pressure isolators and housed in separate polycarbonate cages at constant room temperature (22 °C ± 10%), air humidity (50 ± 20%), and a light/dark cycle of 12 h. Mice had free access to food and water. Standard mouse chow (TD2020SX; Teklad Diets, Madison, WI) was sterilized by irradiation at 25 kGy. GF mice were colonized with EER or human feces by oral gavage (200 μl) and 3–4 mice/cage were housed within gnotobiotic Trexler isolators (EER/GUT-103 experiments and gnotobiotic GUT-108 experiments)^20^ or in sterilized cages with autoclaved food and water (humanized/GUT-108 experiments). Animal use protocols were approved by the Institutional Animal Core and Use Committee of the University of North Carolina at Chapel Hill.

### Inoculation

For treatment 200 µl of diluted human donor stool was applied by oral gavage on day 1 to *Il10*^*−/−*^ mice. The stool was derived from a single healthy donor, Donor-Y, and was previously found to induce moderate to severe colitis in *Il10*^*−/−*^ mice^24^. One gram stool was diluted 100-fold with anaerobic PBS, and vigorously mixed for 5 min under anaerobic conditions. For application of GUT-103 and GUT-108, 300 µl resuspended strain mixture in anaerobic PBS was applied per mouse by oral gavage. GUT-103 and GUT-108 strains were grown individually, subsequently mixed to equal concentrations (cfu/ml), and provided at a dose of 2.0 × 10^+7^ cfu/strain in a total volume of 300 µl. The strain mixture was provided four times via oral gavage on days 15, 17, 22, and 25 (*Il10*^*−/−*^ mice) or on days 1, 3, 8, and 11 (*Il10*^+/eGFP^ mice).

### Fecal collections

Fresh murine feces (2–5 pieces/mouse) were collected and immediately snap-frozen on dry ice and stored at −80 °C. The human fecal sample used for murine fecal transplant was isolated from a healthy volunteer and stored at −80 °C.

### Cell isolation

Colonic tissues were opened longitudinally, washed twice with 1×  PBS, cut into 1 cm pieces and incubated with stirrer for 250 r.p.m. in HBSS (Corning) medium containing 2.5% FBS (Sigma–Aldrich), 1% penicillin–streptomycin (Gibco), 4 mM EDTA (Corning) and 10 mM dithiothreitol (Sigma) for 20 min at 37 °C to remove the epithelial layer. Denuded tissue samples were washed twice with HBSS containing 2.5% FBS and 1% penicillin–streptomycin and incubated with a stirrer at 450 r.p.m. in HBSS containing 2.5% FBS, 0.5 mg/ml of collagenase (Sigma) for 30 min at 37 °C. Cell preparations were filtered through 100-μm nylon mesh to achieve single-cell suspensions. LP cells were purified using a 40-70% discontinuous Percoll gradient (GE Healthcare, 2000 r.p.m., 20 min, room temperature) and washed with HBSS.

### Staining cells for flow cytometry analysis

Single cells were stained for 20 min at 4 °C after FcγRII/III blocking with anti-CD16/CD32 monoclonal antibody. For intracellular staining, cells were restimulated with 50 ng/ml phorbol 12-myristate 13-acetate (PMA, Sigma) and 500 ng/ml ionomycin (Sigma) for 4 h at room temperature with 1 µl/ml protein transport inhibitor (GolgiStop, BD) during the last 3 h. After washing, cells were first surface stained, then fixed for 5 min at 37 °C using PBS containing 4% paraformaldehyde (Electron Microscopy Sciences) and 0.01% Tween 20 (Fisher Scientific), permed using PBS containing 0.1% Triton X-100 (MP Biomedicals), 0.5% BSA (Sigma), 2 mM EDTA (Corning) for 45 min at room temperature, and stained overnight with indicated antibodies. Flow cytometry was performed on a LSRII flow cytometer with FACSDiva software version 6.0 (BD Biosciences). Singlet live CD45^+^ cells were analyzed by FlowJo software version 10 (FlowJo, OR, USA) with the following gating strategy: B cell (B220^+^CD19^+^), CD4^+^ T cell (TCRβ^+^CD3^+^CD4^+^CD8^neg^), macrophage (TCRβ^neg^CD11b^+^CD64^+^), and dendritic cell (TCRβ^neg^CD64^neg^MHCII^+^CD11c^+^). For the GFP-positive gate, GFP-negative colonic LP cells from C57BL/6 wild-type mice were stained with all antibodies used in the experiment as a fluorescence-minus-one control. The flow cytometry gating strategies used to collect the data presented in Fig. 3f and Fig. 5a are provided in Supplementary Fig. 7 and Supplementary Fig. 8, respectively.

The following antibodies were used for flow cytometry: anti-CD16/CD32 monoclonal antibody (Fc block, BD Biosciences, Cat# 553141), Live/Dead fixable dead cell stains (APC-Cy7, Invitrogen, Cat# L10119) at dilution of 1:2400, anti-CD45 antibody (Pacific Orange, Invitrogen, Cat# MCD4530) at dilution of 1:300, anti-B220 antibody (Pacific Blue, Invitrogen, Cat# 48-0452-82) at dilution of 1:200, anti-CD19 antibody (BV605, Biolegend, Cat# 115539) at dilution of 1:300, anti-CD4 antibody (eFluor450, eBioscience, Cat# 48-0041-80) at dilution of 1:200, anti-CD4 antibody (APC, Biolegend, Cat# 100516) at dilution of 1:400, anti-CD3 antibody (PE-Cy5, eBioscience, Cat# 15-0031-81) at dilution of 1:300, anti-TCRb antibody (Alexa Fluor 700, BD Biosciences, Cat# 560705) at dilution of 1:300, anti-CD8 antibody (PE-Cy7, eBioscience, Cat# 25-0081-81) at dilution of 1:300, anti-CD11b antibody (PE-Cy7, BD Biosciences, Cat# 552850) at dilution of 1:300, anti-CD11c antibody (Alexa Fluor 700, BD Biosciences, Cat# 560583) at dilution of 1:400, anti-CD64 antibody (PE, Biolegend, Cat# 139304) at dilution of 1:300, anti-MHC classII antibody (BV650, Biolegend, Cat# 107641) at dilution of 1:300, anti-IFNg antibody (BV421, Biolegend, Cat# 505829) at dilution of 1:300, anti-IL-17a antibody (BV605, Biolegend, Cat# 506927) at dilution of 1:300, anti-Foxp3 antibody (PE, eBioscience, Cat# 12-5773-82) at dilution of 1:300, anti-RORgt antibody (Alexa Fluor 647, BD Biosciences, Cat# 562682) at dilution of 1:300, isotype IgG (PE-Cy5, Biolegend, Cat# 400931) at dilution of 1:300, IgG2 lambda (Alexa Fluor 700, BD Biosciences, Cat# 557985) at dilution of 1:300, IgG2a (APC, BD Biosciences, Cat# 554690) at dilution of 1:300, IgG2a (PE-Cy7, Biolegend, Cat# 400521) at dilution of 1:300, IgG2b (BV650, BD Biosciences, Cat# 563233) at dilution of 1:300, IgG2a (BV605, Biolegend, Cat# 400539) at dilution of 1:300, and IgG2b (Pacific Orange, BD Biosciences, Cat# 553989) at dilution of 1:300.

### Histology and scoring

Fixing, staining, and blind histology scoring were described previously^20^.

### Statistical analysis

GraphPad Prism (version 8) software was used for statistical analysis. For the metabolite analyses, the two-way ANOVA in Graph Pad Prism 8 software was applied to determine the statistical significance of SCFAs and bile acids among groups. Dunnett’s multiple comparisons test was performed to determine whether a specific SCFA or bile acid is significantly different between EER control and other groups. An adjusted *p*-value (calculated by Graph Pad Prism 8 software, version 8.0.0) of <0.05 was considered as significant difference. Results were adjusted based on the amount of stool used.

### Reporting summary

Further information on research design is available in the Nature Research Reporting Summary linked to this article.

## Supplementary information

Supplementary information

Description of Additional Supplementary Files

Supplementary Data 1

Supplementary Data 2

Supplementary Data 3

Supplementary Data 4

Supplementary Data 5

Supplementary Data 6

Supplementary Data 7

Reporting Summary

## Data Availability

The relevant data are available from the authors upon reasonable request. Metagenome sequencing data that support the findings of this study have been deposited in GenBank with the BioProject ID: PRJNA703330. The genome sequences for the GUT-103 consortium strains were downloaded from PATRIC (https://www.patricbrc.org) with the accession codes: 742816.3; 1122216.3; 1120921.3; 1121098.3; 449673.7; 742726.3; 11483.3; 411471.5; 411490.6; 649757.3; 411472.5; 49741.6; 411468.9; 411902.9; 1121114.4; 476272.21; 478749.5. The genome sequences for the novel strains used in the GUT-108 consortium that support the findings of this study have been deposited in GenBank with the accession codes: JABFCE000000000, JABFCF000000000, JABFCG000000000, JABFHK000000000, JABFAG000000000, JABFCH000000000, JABFCI000000000, JABFCJ000000000, JABFCK000000000, JABFCL000000000, and JABFCM000000000. Raw data for the targeted metabolomics are available from Metabolomics Workbench repository, 10.21228/M8XM6R, project ID PR001120. The bacterial strains used in the GUT-103 consortium are commercially available from ATCC (https://www.ATCC.org) and DSMZ (https://www.DSMZ.de). The bacterial strains used in the GUT-108 consortium can be obtained from the corresponding author through a Material Transfer Agreement with the restriction that they can only be used for academic research purposes. Source data are provided with this paper.

## Source data

Source Data
