# Supplementary material for: Rationally designed bacterial consortia to treat chronic immune-mediated colitis and restore intestinal homeostasis
Source: Nat Commun. 2021 May 28;12:3105. doi: 10.1038/s41467-021-23460-x (PMC8163890; doi:10.1038/s41467-021-23460-x)
Supplement: Supplementary file 1 — Supplementary information [file 41467_2021_23460_MOESM1_ESM.pdf]

SUPPLEMENTARY INFORMATION

TITLE

Rationally designed bacterial consortia to treat chronic immune-mediated colitis and restore intestinal homeostasis

AUTHORS

Daniel van der Lelie[1]*, Akihiko Oka[2,3], Safiyh Taghavi[1], Junji Umeno[2,4], Ting- Jia Fan[1], Katherine E. Merrell[1], Sarah D. Watson[1], Lisa Ouellette[1], Bo Liu[2], Muyiwa Awoniyi[2], Yunjia Lai[5], Liang Chi[5], Kun Lu[5], Christopher S. Henry[1], R. Balfour Sartor[2]

[1] Gusto Global LLC. 400 Park Offices Drive (400 The Frontier, Suite 104), Morrisville, NC 27560, USA, [2] Departments of Medicine, Microbiology and Immunology, Center for Gastrointestinal Biology and Disease, University of North Carolina at Chapel Hill, Chapel Hill, NC 27599, USA, [3] Department of Internal Medicine II, Shimane University Faculty of Medicine, 89-1 Izumo, 693-8501 Shimane, Japan, [4] Department of Medicine and Clinical Science, Graduate School of Medical Sciences, Kyushu University, Maidashi 3-1-1, Higashi-Ku, Fukuoka, Fukuoka 812-8582, Japan, [5] Department of Environmental Sciences and Engineering, Gillings School of Public Health, University of North Carolina at Chapel Hill, Chapel Hill, NC 27599, USA

*To whom correspondence should be addressed: dvanderlelie@gustoglobal.com.

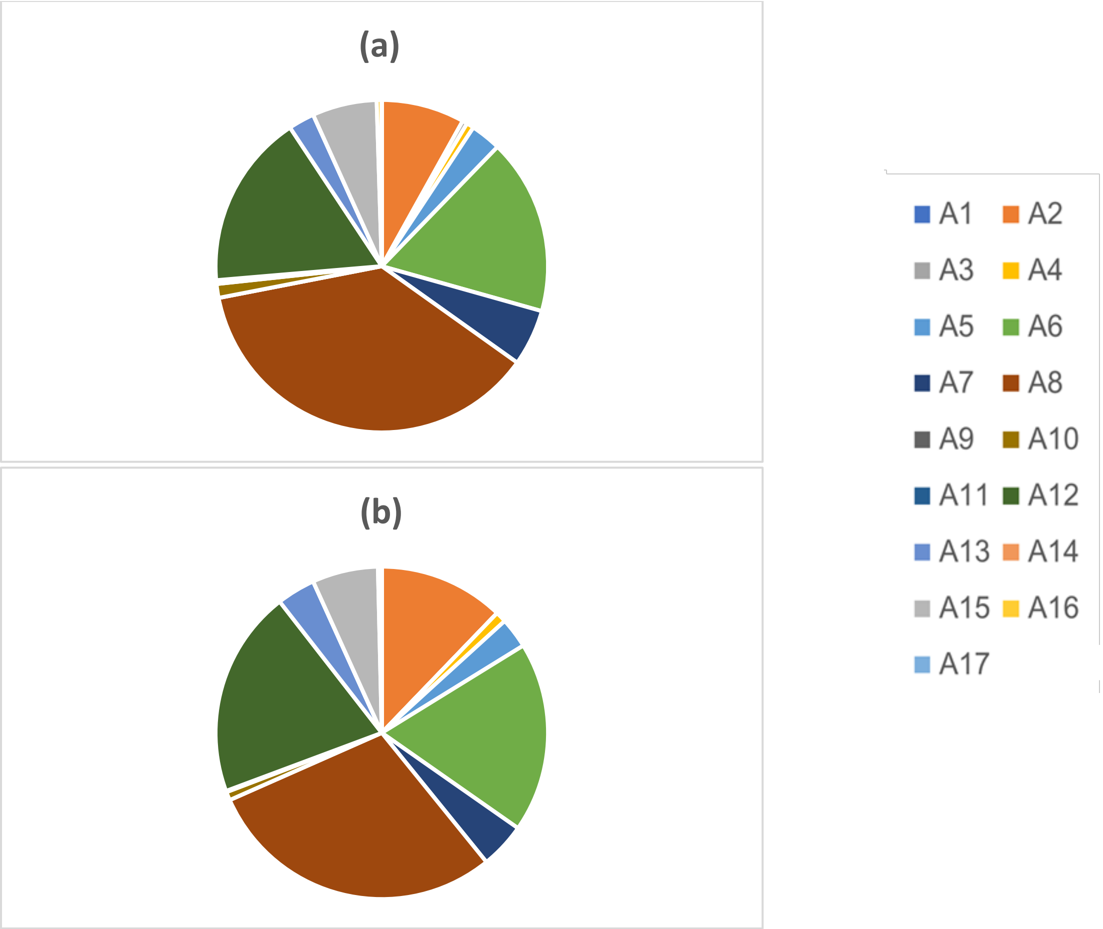

Supplementary Fig. 1a and Fig. 1b. Microbial composition of gnotobiotic 129 wild type (**a**) and gnotobiotic 129 IL10 $^{-/-}$ mice (**b**) inoculated with GUT-103. The microbial composition of fecal pellets was determined two weeks after inoculation using qPCR on the species specific *rpoB* gene.

Strain legend: A1: *Megamonas hypermegale* DSM1672; A2 *Bacteroides stercoris* DSM19555; A3: *Anaerostipes hadrus* DSM3319; A4: *Clostridium symbiosum* ATCC14940; A5: *Clostridium boltea* ATCC BAA-613; A6: *Blautia producta* DSM2950; A7: *Clostridium scindens* ATCC35704; A8: *Akkermansia muciniphila* ATCC BAA-835; A9: *Marvinbryantia formatexigens* DSM14469; A10: *Megamonas funiformis* DSM19343; A11: *Acidaminococcus intestini* DSM21505; A12: *Bacteroides massiliensis* DSM17679; A13: *Barnesiella intestinihominis* DSM21032; A14: *Faecalibacterium prausnitzii* DSM17677; A15: *Subdoligranulum variabile* DSM15176; A16: *Anaerostipes caccae* DSM14662; A17: *Blautia hydrogenotrophica* DSM10507.

Code	Strain	Relative presence in 129 wild type mice (n=3)	Relative presence in 129 IL10 $^{-/-}$ mice (n=5)
A1	*Megamonas hypermegale* DSM1672	0.000% ±0.000%	3.822% ±5.392%
A2	*Bacteroides stercoris* DSM19555	9.934% ±1.607%	8.990% ±2.042%
A3	*Anaerostipes hadrus* DSM3319	0.371% ±0.331%	0.300% ±0.273%
A4	*Clostridium symbiosum* ATCC14940	0.710% ±0.157%	0.872% ±0.267%
A5	*Clostridium boltea* ATCC BAA-613	2.887% ±0.556%	2.605% ±0.290%
A6	*Blautia producta* DSM2950	16.811% ±6.714%	19.508% ±1.471%
A7	*Clostridium scindens* ATCC35704	4.830% ±1.055%	4.929% ±0.862%
A8	Akkermansia muciniphila ATCC BAA-835	39.486% ±11.203%	29.149% ±1.377%
A9	*Marvinbryantia formatexigens* DSM14469	0.000% ±0.000%	0.000% ±0.000%
A10	*Megamonas funiformis* DSM19343	0.453% ±0.775%	1.687% ±1.153%
A11	*Acidaminococcus intestini* DSM21505	0.205% ±0.136%	0.212% ±0.160%
A12	*Bacteroides massiliensis* DSM17679	16.496% ±2.203%	19.813% ±2.215%
A13	*Barnesiella intestinihominis* DSM21032	2.877% ±0.487%	4.523% ±0.771%
A14	*Faecalibacterium prausnitzii* DSM17677	0.000% ±0.000%	0.000% ±0.000%
A15	*Subdoligranulum variabile* DSM15176	4.603% ±1.639%	7.139% ±1.514%
A16	*Anaerostipes caccae* DSM14662	0.330% ±0.139%	0.268% ±0.081%
A17	*Blautia hydrogenotrophica* DSM10507	0.000% ±0.000%	3.822% ±5.392%

Supplementary Table 1. Data on microbial composition of gnotobiotic 129 wild type and gnotobiotic 129 IL10 $^{-/-}$ mice inoculated with GUT-103. The microbial composition of fecal pellets was determined two weeks after inoculation using qPCR on the species specific *rpoB* gene. The STDEV function of excel to calculate the standard deviation of the samples. The data from this table were used to create Supplementary Fig. 1a and Supplementary Fig. 1b.

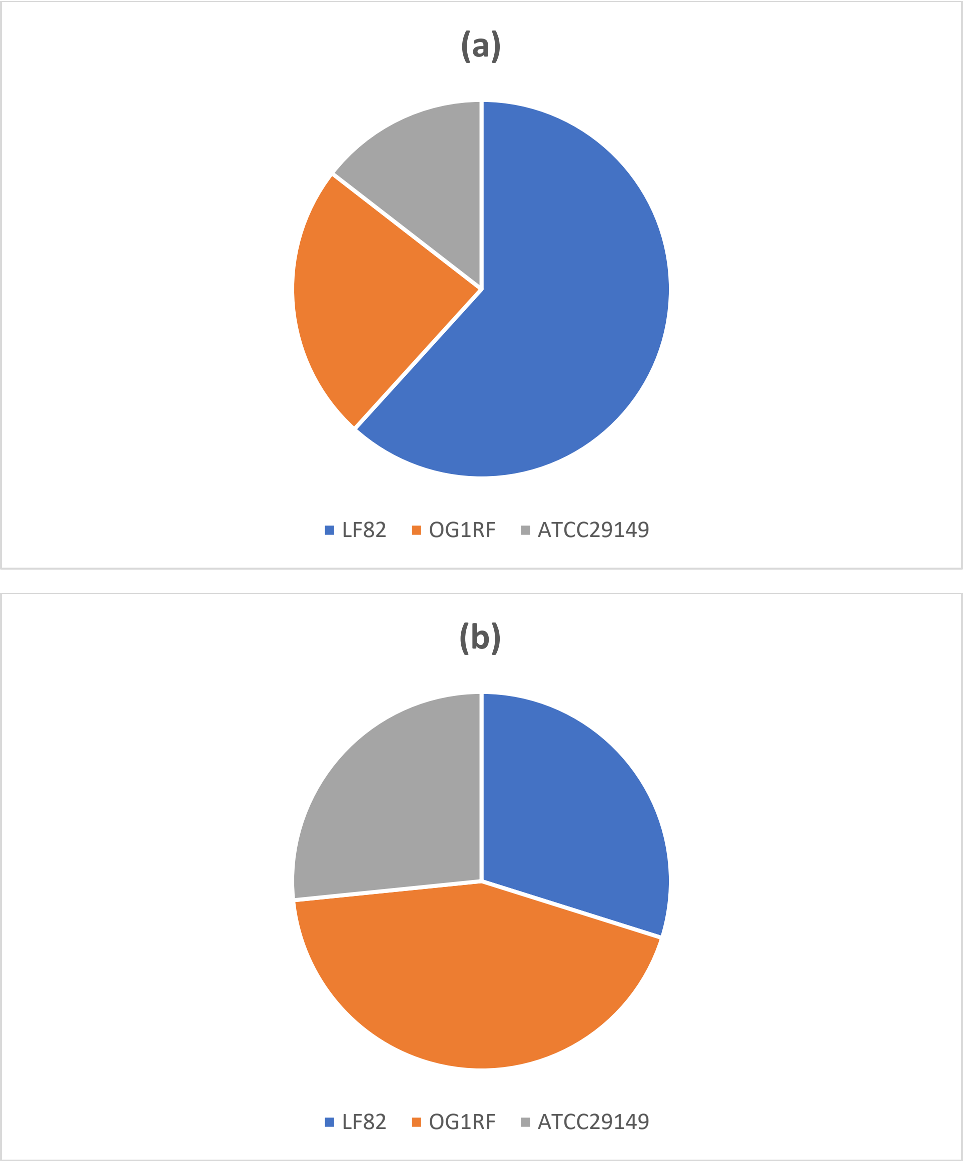

Supplementary Fig. 2a and Fig. 2b. Microbial composition of gnotobiotic 129 wild type (**a**) and gnotobiotic 129 IL10 $^{-/-}$ mice (**b**) inoculated with the EER consortium comprised of *Escherichia coli LF82, Enterococcus faecalis OG1RF* and *Ruminococcus gnavus ATCC29149*. The microbial composition of fecal pellets was determined two weeks after inoculation using qPCR on the species specific *rpoB* gene.

Strain	Relative presence in 129 wild type mice (n=3)	Relative presence in 129 IL10 $^{-/-}$ mice (n=3)
Escherichia coli LF82	61.731% ±6.733%	29.853% ±19.745%
Enterococcus faecalis OG1RF	33.763%±5.148%	43.538% ±19.057%
Ruminococcus gnavus ATCC29149	14.506% ±1.585%	26.610% ±3.825%

Supplementary Table 2. Data on microbial composition of gnotobiotic 129 wild type (A) and gnotobiotic 129 IL10 $^{-/-}$ mice (B) inoculated with the EER consortium. The microbial composition of fecal pellets was determined two weeks after inoculation using qPCR on the species specific *rpoB* gene. The STDEV function of excel to calculate the standard deviation of the samples. The data from this table were used to create Supplementary Fig. 2a and Supplementary Fig. 2b.

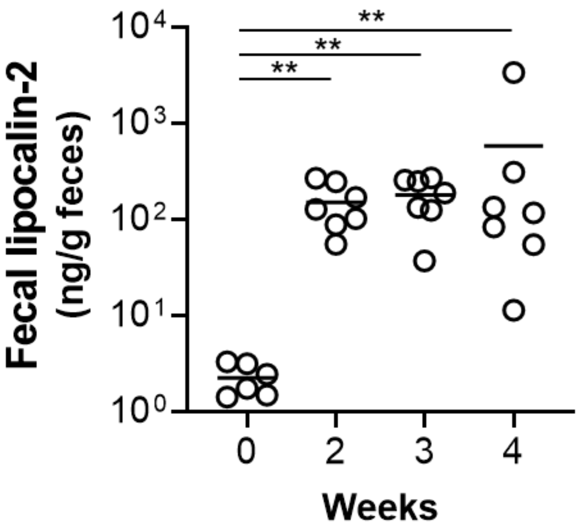

Supplementary Fig. 3: Progression of fecal lipocalin-2 levels after inoculation of germ-free (GF) *Il10*$^{-/-}$ mice with the EER consortium comprised of *Escherichia coli* LF82, *Enterococcus faecalis* OG1RF and *Ruminococcus gnavus* ATCC29149.

Germ-free (GF) *Il10*$^{-/-}$ mice were inoculated with EER consortium on T0. Feces were collected and analyzed by fecal lipocalin-2 ELISA at 0, 2, 3 and 4 weeks after EER-inoculation. Bars show mean. **P < 0.01. Mann Whitney Unpaired t-test.

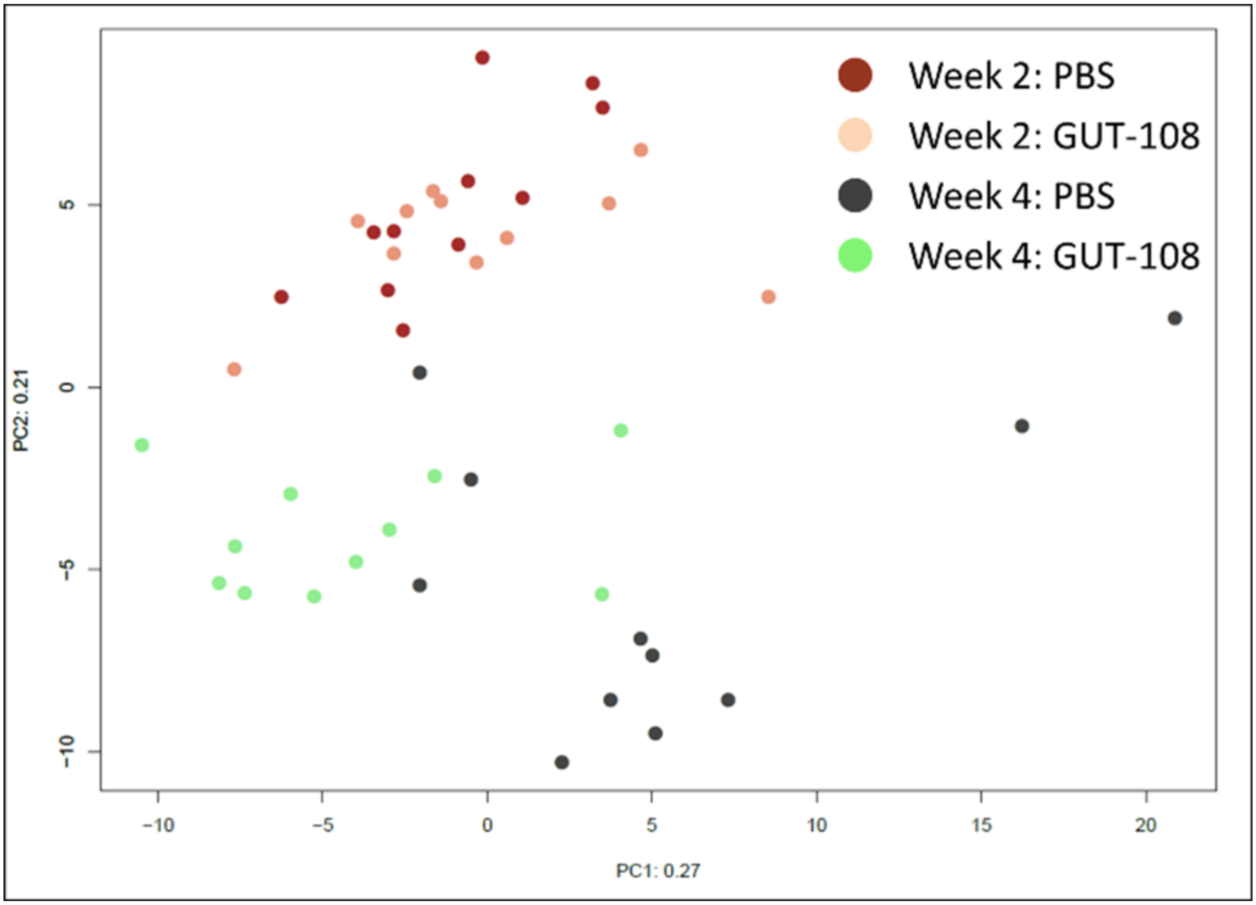

Supplementary Fig. 4: PCA analysis based on genus relative abundance as determined by analysis of metagenomic sequencing reads with Kaiju (http://kaiju.binf.ku.dk/). On day 1, mice were inoculated with a human stool previously verified to induce aggressive colitis in gnotobiotic *Il10*$^{-/-}$ mice. Therapeutic application of GUT-108 or PBS control started after 2 weeks. Dots represent the gut microbiome composition of individual mice.

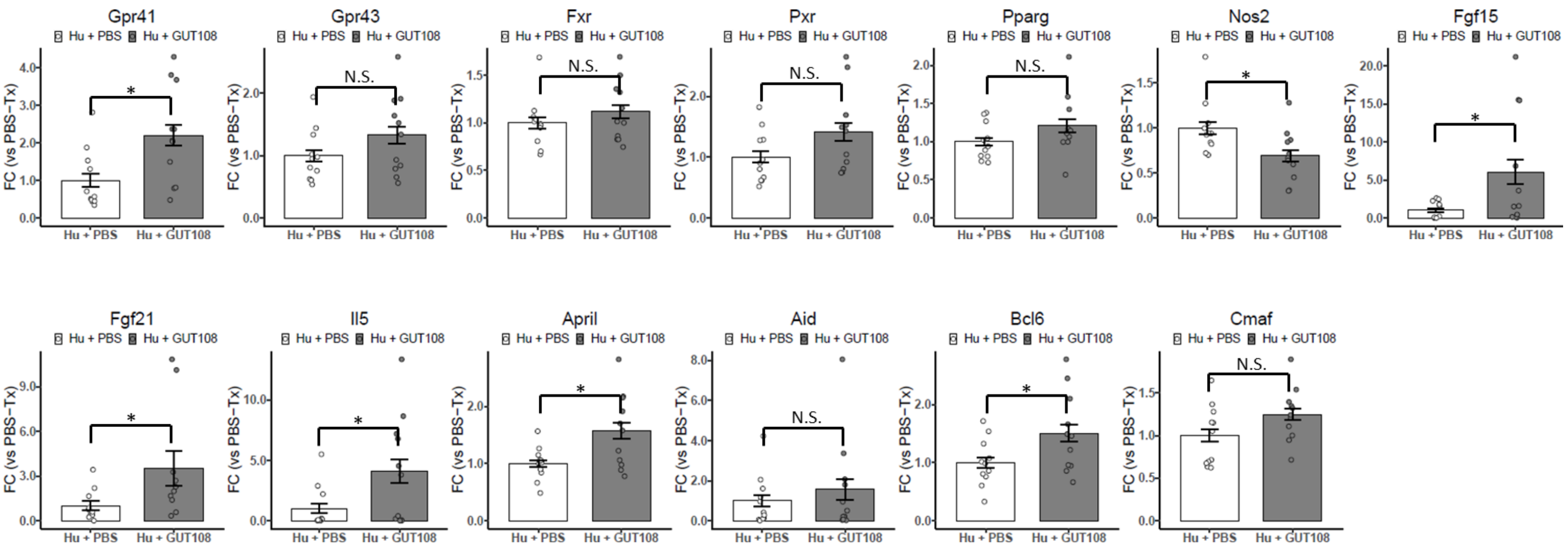

Supplementary Fig. 5: Expression of metabolite sensors and mediators (*Gpr41, Gpr43, Fxr, Pxr, Pparg, Fgf15, Fgf21*), and mediators and pathways involved in the differentiation of immune cells including Treg and Breg cells (*cMaf, Il5, April, Aid, Bcl6*) Expression levels were determined by RT-Q-PCR and presented as fold change (FC) of expression after therapeutic application of GUT-108 compared to expression after PBS treatment. Hu+GUT-108 refers to humanized *Il10*$^{-/-}$ mice treated with GUT-108 in a therapeutic protocol; Hu+PBS refers to humanized *Il10*$^{-/-}$ mice that received PBS as a placebo control. Bar indicates Mean ± SE. Two-way ANOVA

and adjusted P-value were calculated by the multiple comparisons test. *: adjusted P<0.05; N.S.: not significant. N = 11. Source data are provided as a Source Data file.

Specificity of *rpoB* primers for quantitative PCR on GUT-103 and GUT-108 strains.

Materials and Methods

Primers were designed as described in the manuscript with their sequences provided in Supplementary Data 7: RpoB gene primers for GUT-103 and GUT-108 composition studies by Q-PCR.

Q-PCR were performed with QuantStudio3 (Thermo Fisher Scientific, PA, USA) using SYBR No-ROX reagents (Bioline, TN, USA) and 10ng of genomic DNA per strain with the following PCR settings: 95°C, 3 min; 40 cycles of (95°C, 5 sec; 60°C, 20 sec); melting curve analysis: 95°C, 15 sec; 60°C, 15 sec; 95°C, 15 sec. The data were created by comparative Ct method (2^{-Ct}). Melting curve analysis confirmed the presence of single products with expected melting temperatures.

Results

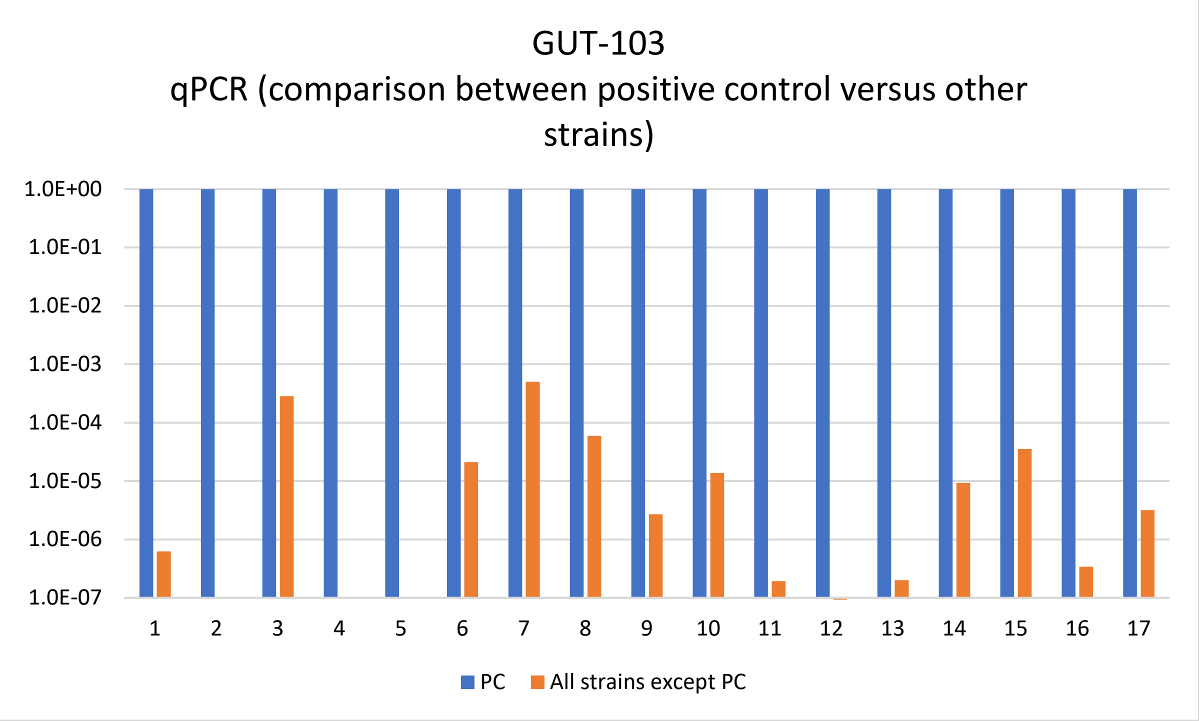

Supplementary Fig. 6a. Specificity of *rpoB* primers for quantitative RT-PCR on GUT-103 strains. Blue bars: Q-PCR positive control on the specific strain (PC); Orange bars: PCR negative control on a mixture of genomic DNAs from all strains except the positive control for the specific strain. The signal of the negative control was expressed as its strength compared to the positive control (set at 1.0). 1: *Megamonas hypermegale* DSM1672; 2 *Bacteroides stercoris* DSM19555; 3: *Anaerostipes hadrus* DSM3319; 4: *Clostridium symbiosum* ATCC14940; 5: *Clostridium boltea* ATCC BAA-613; 6: *Blautia producta* DSM2950; 7: *Clostridium scindens* ATCC35704; 8: *Akkermansia muciniphila* ATCC BAA-835; 9: *Megamonas funiformis* DSM19343; 10: *Acidaminococcus intestini* DSM21505; 11: *Bacteroides massiliensis* DSM17679; 12: *Barnesiella intestinihominis* DSM21032; 13: *Faecalibacterium prausnitzii* DSM17677; 14: *Subdoligranulum variabile* DSM15176; 15: *Anaerostipes caccae* DSM14662; 16: *Blautia hydrogenotrophica* DSM10507; 17: *Marvinbryantia formatexigens* DSM14469.

For each of the GUT-103 strain PCR primer pairs, the strength of the non-specific amplification signal was >1000x less than the amplification signal for its positive PCR control.

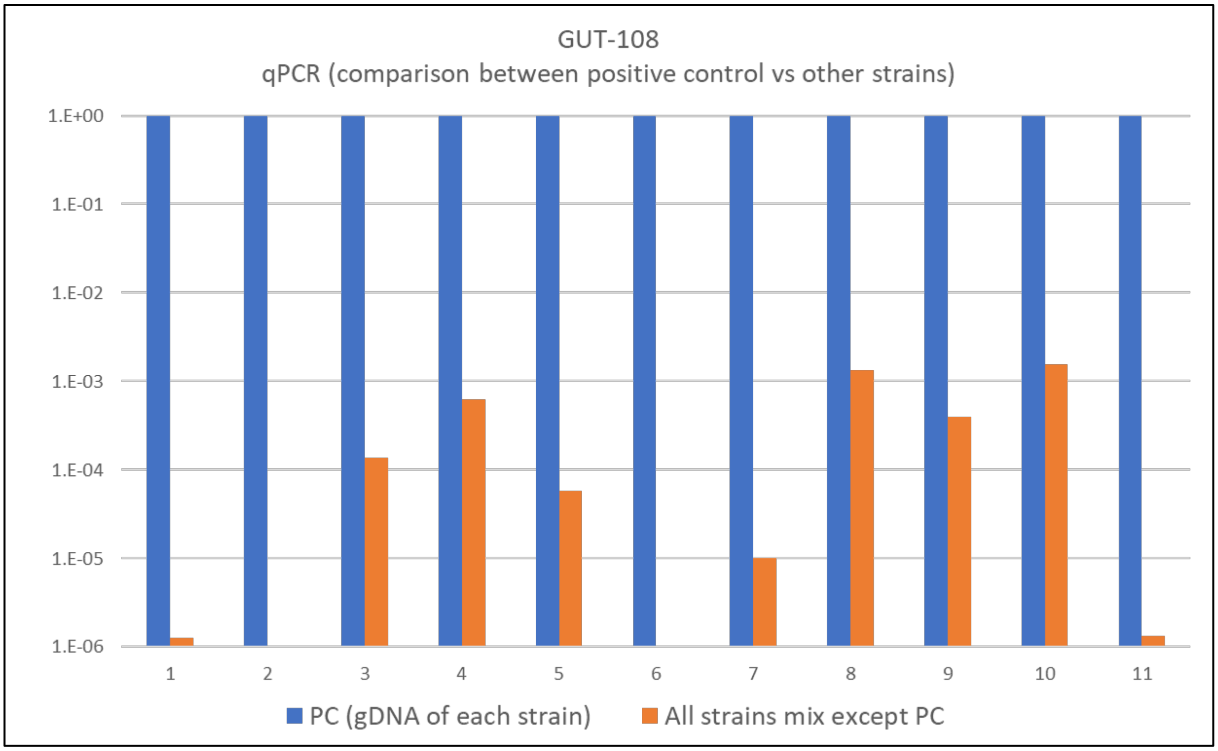

Supplementary Fig. 6b. Specificity of *rpoB* primers for quantitative RT-PCR on GUT-108 strains. Blue bars: Q-PCR positive control on the specific strain (PC); Orange bars: PCR negative control on a mixture of genomic DNAs from all strains except the positive control for the specific strain. The signal of the negative control was expressed as its strength compared to the positive control (set at 1.0). 1: *Bacteroides xylanisolvens* GGCC_0124; 2: *Bacteroides uniformis* GGCC_0301; 3: *Clostridium butyricum* GGCC_0151; 4: *Eubacterium callanderi* GGCC_0197; 5: *Barnesiella* sp. GGCC_0306; 6: *Clostridium symbiosum* GGCC_0272; 7: *Bittarella massiliensis* GGCC_0305; 8: *Extibacter* sp. GGCC_0201; 9: *Clostridium scindens* GGCC_0168; 10: *Akkermansia* sp. GGCC_0220; 11: *Intestinimonas butyriciproducens* GGCC_0179.

For each of the GUT-108 strain PCR primer pairs, the strength of the non-specific amplification signal was approximately 1000x less or smaller than the amplification signal for its positive PCR control.

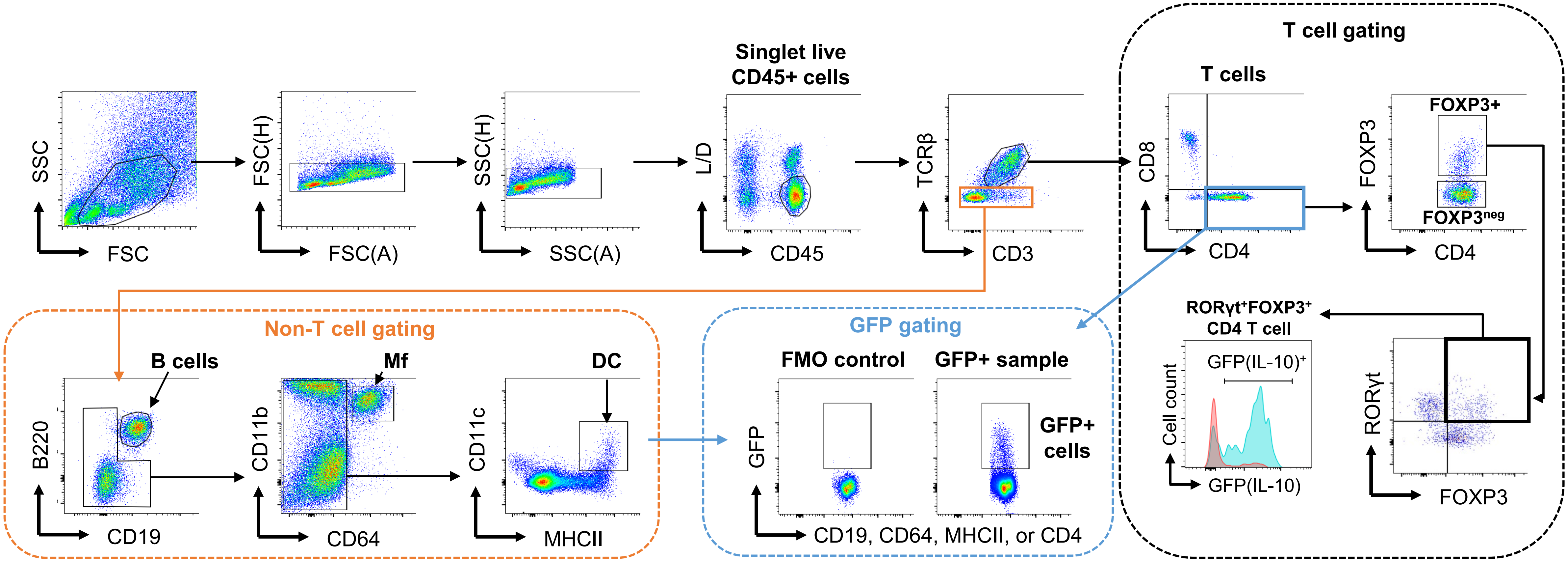

Supplementary Fig. 7. Sequential flowcytometry gating/sorting strategy in support of Figure 3f to examine the level of induction of IL-10-producing T and B cells, dendritic cells (DC) and macrophages (Mf), and different types of IL-10+ regulatory T cells.
FSC: forward scatter, SSC: side scatter, A: area, H: height, Mf: macrophage, DC: dendritic cell, FMO: fluorescence-minus-one.

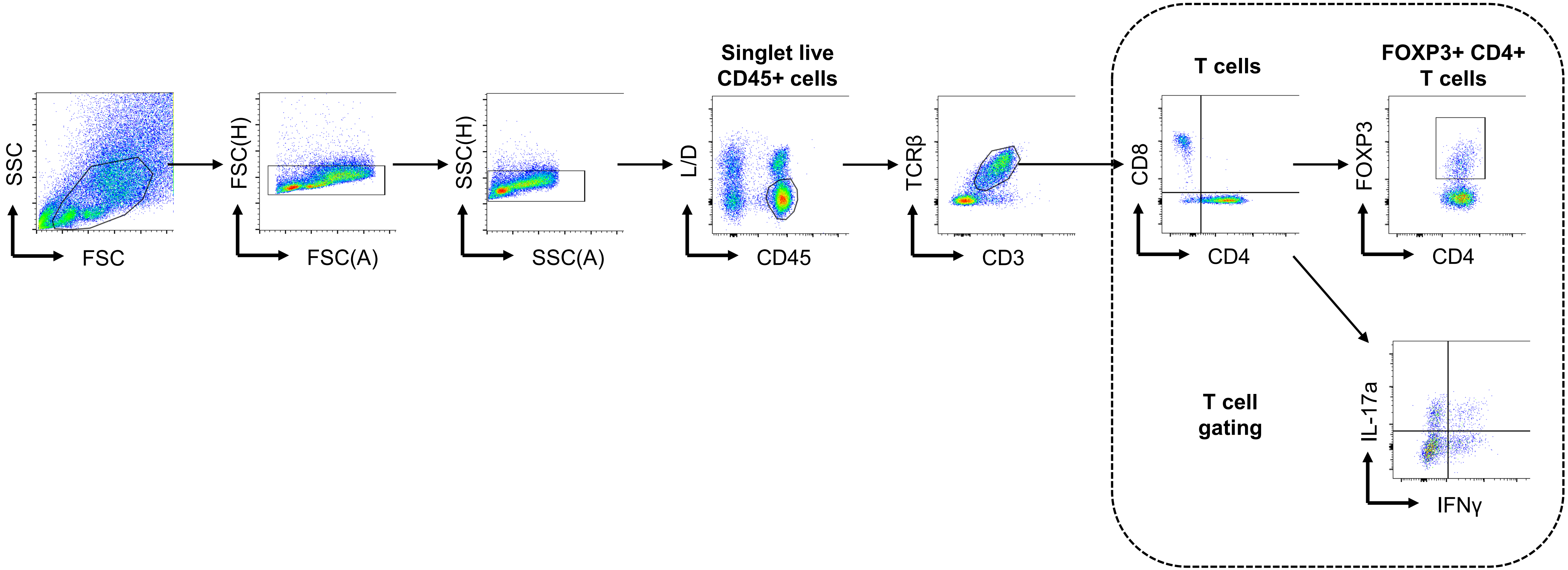

Supplementary Fig. 8. Sequential flowcytometry gating/sorting strategy in support of Figure 5a to examine the level of induction of colonic lamina propria effector CD4+ T cells, including Th1 and Th17 cells that produce IFNγ and/or IL-17α.
FSC: forward scatter, SSC: side scatter, A: area, H: height, Mf: macrophage, DC: dendritic cell, FMO: fluorescence-minus-one.

species	Ratio PBS W4/PBS W2	Ratio GUT-108 W4/PBS W2
Shigella sonnei	193.2327	1.645933
Shigella dysenteriae	179.4068	1.951144
Bacteroides eggerthii CAG:109	169.9554	1.973646
Shigella flexneri	158.4821	1.894698
Alistipes sp. CAG:29	120.2647	21.69084
Escherichia coli	119.5442	2.106232
Collinsella sp. CAG:166	82.11031	99.84399
Collinsella sp. 4_8_47FAA	53.04236	86.01151
Faecalicoccus pleomorphus	52.59167	98.85213
Klebsiella pneumoniae	45.00416	3.049834
Alistipes finegoldii	38.57147	8.770555
Merdimonas faecis	36.54296	37.55588
Collinsella aerofaciens	27.19046	50.75322
Alistipes sp. CAG:53	25.39727	13.97785
Salmonella enterica	19.29118	1.774586
Alistipes sp. HGB5	18.05325	2.332046
Anaerotruncus colihominis	15.54019	18.05248
Alistipes shahii	11.70823	7.377604
Bacteroides intestinalis	9.878536	6.617156
Bacteroides sp. 14(A)	9.800727	2.171663
Bacteroides sp. 41_26	9.558624	16.10986
Bacteroides cellulosilyticus	9.41403	2.396796
Alistipes sp. 56_sp_Nov_56_25	8.731694	6.741683
Bacteroides intestinalis CAG:564	8.714981	10.84117

Supplementary Table 3. Fold expansion of the gut pathobiome over time for mice that were inoculated with a human stool previously verified to induce aggressive colitis in gnotobiotic *Il10*$^{-/-}$ mice. Therapeutic application of GUT-108 or PBS control started after 2 weeks. The fold expansion over the two-week period between week 2 and week 4 was calculated as the ratio of the percentage of the total community composition for specific bacterium at week 4 over that at week 2. The results are shown for the 24 species whose relative percentage in the gut microbiome of PBS treated mice was found to be the most increased over the two-week period. Bacteria highlighted in red represent well-known species of opportunistic pathogens belonging to the *Enterobacteriaceae* family.

In animals receiving therapeutic application of GUT-108, expansion of opportunistic pathogenic bacteria belonging to the *Enterobacteriaceae* family ranged from 1.65 (*Shigella sonnei*) to 3.05 (*Klebsiella pneumoniae*). On the other hand, the expansion of opportunistic pathogenic bacteria belonging to the *Enterobacteriaceae* family ranged from 19.29 (*Salmonella enterica*) to 193.23 (*Shigella sonnei*) for animals receiving PBS as therapeutic application. The results show that over time the normal microbiota from a healthy donor are altered in an inflammatory environment as found in *Il10*$^{-/-}$ mice that received PBS as control treatment, with a significant increase in opportunistic pathogenic bacteria belonging to the *Enterobacteriaceae* family. On the other hand, *Il10*$^{-/-}$ mice that received therapeutic treatment with GUT-108 showed considerably less expansion of their gut pathobiome.
