# Supplementary material for: Rationally designed bacterial consortia to treat chronic immune-mediated colitis and restore intestinal homeostasis
Source: Nat Commun. 2021 May 28;12:3105. doi: 10.1038/s41467-021-23460-x (PMC8163890; doi:10.1038/s41467-021-23460-x)
Supplement: Supplementary file 2 — Description of Additional Supplementary Files [file 41467_2021_23460_MOESM2_ESM.docx]

File Name: Supplementary Data 1
Description: Comparative analysis of the annotated genomes of *Akkermansia* species. After annotation, the genomes were compared for the presence of auxotrophies, therapeutic functions (bile acid conversion, synthesis of butyrate and propionate) and functions important for niche competition (iron uptake and lantibiotic synthesis). Results for whole genome DNA average nucleotide identity (ANI) analysis for *Akkermansia* species are also presented.

File Name: Supplementary Data 2
Description: Comparative analysis of the annotated genomes of *Bacteroides uniformis*. After annotation, the genomes were compared for the presence of auxotrophies, therapeutic functions (bile acid conversion, synthesis of butyrate and propionate) and functions important for niche competition (iron uptake and lantibiotic synthesis). Results for whole genome DNA average nucleotide identity (ANI) analysis for *Bacteroides uniformis* are also presented.

File Name: Supplementary Data 3
Description: Comparative analysis of the annotated genomes of *Eubacterium limosum*. After annotation, the genomes were compared for the presence of auxotrophies, therapeutic functions (bile acid conversion, synthesis of butyrate and propionate) and functions important for niche competition (iron uptake and lantibiotic synthesis). Results for whole genome DNA average nucleotide identity (ANI) analysis for *Eubacterium limosum* are also presented.

File Name: Supplementary Data 4
Description: Comparative analysis of the annotated genomes of *Faecalibacterium* species. After annotation, the genomes were compared for the presence of auxotrophies, therapeutic functions (bile acid conversion, synthesis of butyrate and propionate) and functions important for niche competition (iron uptake and lantibiotic synthesis). Results for whole genome DNA average nucleotide identity (ANI) analysis for *Faecalibacterium* species are also presented.

File Name: Supplementary Data 5
Description: Description of the GUT-103 functional characteristics, auxotrophy reactions, flux balance analysis and strain interaction table to predict the growth of the individual GUT-103 strains and the GUT-103 consortium under defined conditions.

File Name: Supplementary Data 6
Description: Description: Description of the GUT-108 functional characteristics, auxotrophy reactions, flux balance analysis and strain interaction table to predict the growth of the individual GUT-108 strains and the GUT-108 consortium under defined conditions.

File Name: Supplementary Data 7
Description: Overview of the PCR primers used in this study.
