# Supplementary material for: Rationally designed bacterial consortia to treat chronic immune-mediated colitis and restore intestinal homeostasis
Source: Nat Commun. 2021 May 28;12:3105. doi: 10.1038/s41467-021-23460-x (PMC8163890; doi:10.1038/s41467-021-23460-x)
Supplement: Supplementary file 10 — Reporting Summary [file 41467_2021_23460_MOESM10_ESM.pdf]

nature research

Corresponding author(s): Daniel van der Lelie

Last updated by author(s): Apr 28, 2021

Reporting Summary

Nature Research wishes to improve the reproducibility of the work that we publish. This form provides structure for consistency and transparency in reporting. For further information on Nature Research policies, see our Editorial Policies and the Editorial Policy Checklist.

Statistics

For all statistical analyses, confirm that the following items are present in the figure legend, table legend, main text, or Methods section.

n/a	Confirmed	
☐	☒	The exact sample size (*n*) for each experimental group/condition, given as a discrete number and unit of measurement
☐	☒	A statement on whether measurements were taken from distinct samples or whether the same sample was measured repeatedly
☐	☒	The statistical test(s) used AND whether they are one- or two-sided<br>*Only common tests should be described solely by name; describe more complex techniques in the Methods section.*
☒	☐	A description of all covariates tested
☒	☐	A description of any assumptions or corrections, such as tests of normality and adjustment for multiple comparisons
☐	☒	A full description of the statistical parameters including central tendency (e.g. means) or other basic estimates (e.g. regression coefficient) AND variation (e.g. standard deviation) or associated estimates of uncertainty (e.g. confidence intervals)
☐	☒	For null hypothesis testing, the test statistic (e.g. *F*, *t*, *r*) with confidence intervals, effect sizes, degrees of freedom and *P* value noted<br>*Give P values as exact values whenever suitable.*
☒	☐	For Bayesian analysis, information on the choice of priors and Markov chain Monte Carlo settings
☒	☐	For hierarchical and complex designs, identification of the appropriate level for tests and full reporting of outcomes
☒	☐	Estimates of effect sizes (e.g. Cohen's *d*, Pearson's *r*), indicating how they were calculated

Our web collection on statistics for biologists contains articles on many of the points above.

Software and code

Policy information about availability of computer code

Data collection	Sequencing was performed using a 2×150 paired-end configuration; image analysis and base calling were conducted by the HiSeq Control Software (v2.0.12) on the HiSeq instrument. Sequencing reads were trimmed, and mouse reads were filtered out using Trimmomatic (version v0.39). After trimming, sequencing reads were assembled with SPAdes (version 3.13.0).<br>Species abundance was determined with Kaiju (version v1.7.2).<br>Q-PCR were performed with QuantStudio 3 (Thermo Fisher Scientific, PA, USA).<br>Flow cytometry was performed on a LSRII flow cytometer with FACSDiva software version 6.0 (BD Biosciences) and further analyzed using FlowJo software version 10 (FlowJo, OR, USA).
Data analysis	Genome sequences for GUT-108 strains or reference strains, downloaded from GenBank files (National Center for Biotechnology Information, NCBI) or Pathosystems Resource Integration Center (PATRIC) were consistently annotated using Prokka (version 1.14-dev), the RAST server, antiSMASH (version 5.1.1) and Blast against custom-built protein databases. Models were reconstructed, gapfilled, and Flux Balance Analysis (66) (FBA) was run using the KBase fbatools module v2.0, which is currently deployed in beta mode in the KBase narrative interface (https://narrative.kbase.us/). Reconstruction was performed using the "Build metabolic model" app; gapfilling was performed using the "Gapfill Metabolic Model" app; all flux balance analysis was performed using the "Run Flux Balance Analysis" app; community models were assembled using the "Merge Metabolic Models into Community Model" app; and community flux balance analysis was performed using the "Run Flux Balance Analysis" app. As a last step, all models were manually curated and refined prior to final analysis. Auxotrophies were determined by reviewing the Pathway table to determine if the compounds were created via a reaction or combination of reactions.<br>GraphPad Prism (version 8) software was used for statistical analysis.

For manuscripts utilizing custom algorithms or software that are central to the research but not yet described in published literature, software must be made available to editors and reviewers. We strongly encourage code deposition in a community repository (e.g. GitHub). See the Nature Research guidelines for submitting code & software for further information.

Data

Policy information about availability of data

All manuscripts must include a data availability statement. This statement should provide the following information, where applicable:

- Accession codes, unique identifiers, or web links for publicly available datasets
- A list of figures that have associated raw data
- A description of any restrictions on data availability

The relevant data are available from the authors upon reasonable request. Metagenome sequencing data that support the findings of this study have been deposited in GenBank with the BioProject ID: PRJNA703330 (http://www.ncbi.nlm.nih.gov/bioproject/703330).
The genome sequences for the GUT-103 consortium strains were downloaded from PATRIC (https://www.patricbrc.org) with the accession codes: 742816.3; 1122216.3; 1120921.3; 1121098.3; 449673.7; 742726.3; 11483.3; 411471.5; 411490.6; 649757.3; 411472.5; 49741.6; 411468.9; 411902.9; 1121114.4; 476272.21; 478749.5.
The genome sequences for the novel strains used in the GUT-108 consortium that support the findings of this study have been deposited in GenBank with the accession codes: JABFCE000000000, JABFCF000000000, JABFCG000000000, JABFHK000000000, JABFAG000000000, JABFCH000000000, JABFCI000000000, JABFCJ000000000, JABFCK000000000, JABFCL000000000 and JABFCM000000000.
Raw data for the targeted metabolomics are available from Metabolomics Workbench repository, http://dx.doi.org/10.21228/M8XM6R, project ID PR001120.
The bacterial strains used in the GUT-103 consortium are commercially available from ATCC (https://www.ATCC.org) and DSMZ (https://www.DSMZ.de). The bacterial strains used in the GUT-108 consortium can be obtained from the corresponding author through a Material Transfer Agreement with the restriction that they can only be used for academic research purposes.
Source Data are provided with this paper.

Field-specific reporting

Please select the one below that is the best fit for your research. If you are not sure, read the appropriate sections before making your selection.

[x] Life sciences [ ] Behavioural & social sciences [ ] Ecological, evolutionary & environmental sciences

For a reference copy of the document with all sections, see nature.com/documents/nr-reporting-summary-flat.pdf

Life sciences study design

All studies must disclose on these points even when the disclosure is negative.

Sample size	A formal sample size calculation was not performed since these were novel exploratory studies. Sample size was empirically determined to optimize numbers necessary for statistical significance based on our previous studies [ref# 19-23, 41] using IL10 -/- mice and the similar disease models (minimum of 5 mice/group). We used 11 mice/group as a very conservative number to ensure statistical significance and reproducibility.
Data exclusions	No data were excluded from the study.
Replication	The GUT-103 consortium was tested in two different mouse models; the GUT-108 consortium was tested in 2 different mouse models. All attempts at replication were successful.
Randomization	The animals were randomly selected for each treatment group.
Blinding	The level of inflammation (colitis) was determined by blind histology scoring.

Reporting for specific materials, systems and methods

We require information from authors about some types of materials, experimental systems and methods used in many studies. Here, indicate whether each material, system or method listed is relevant to your study. If you are not sure if a list item applies to your research, read the appropriate section before selecting a response.

Materials & experimental systems

n/a	Involved in the study	
[ ]	[x]	Antibodies
[x]	[ ]	Eukaryotic cell lines
[x]	[ ]	Palaeontology and archaeology
[ ]	[x]	Animals and other organisms
[ ]	[x]	Human research participants
[x]	[ ]	Clinical data
[x]	[ ]	Dual use research of concern

Methods

n/a	Involved in the study	
[x]	[ ]	ChIP-seq
[ ]	[x]	Flow cytometry
[x]	[ ]	MRI-based neuroimaging

Antibodies

Antibodies used	anti-CD16/CD32 monoclonal antibody (Fc block, BD Biosciences, Cat# 553141)<br>Live/Dead fixable dead cell stains (APC-Cy7, Invitrogen, Cat# L10119) at dilution of 1:2400<br>anti-CD45 antibody (Pacific Orange, Invitrogen, Cat# MCD4530) at dilution of 1:300<br>anti-B220 antibody (Pacific Blue, Invitrogen, Cat# 48-0452-82) at dilution of 1:200<br>anti-CD19 antibody (BV605, Biolegend, Cat# 115539) at dilution of 1:300<br>anti-CD4 antibody (eFluor450, eBioscience, Cat# 48-0041-80) at dilution of 1:200<br>anti-CD4 antibody (APC, Biolegend, Cat# 100516) at dilution of 1:400<br>anti-CD3 antibody (PE-Cy5, eBioscience, Cat# 15-0031-81) at dilution of 1:300<br>anti-TCRb antibody (Alexa Fluor 700, BD Biosciences, Cat# 560705) at dilution of 1:300<br>anti-CD8 antibody (PE-Cy7, eBioscience, Cat# 25-0081-81) at dilution of 1:300<br>anti-CD11b antibody (PE-Cy7, BD Biosciences, Cat# 552850) at dilution of 1:300<br>anti-CD11c antibody (Alexa Fluor 700, BD Biosciences, Cat# 560583) at dilution of 1:400<br>anti-CD64 antibody (PE, Biolegend, Cat# 139304) at dilution of 1:300<br>anti-MHC classII antibody (BV650, Biolegend, Cat# 107641) at dilution of 1:300<br>anti-IFNg antibody (BV421, Biolegend, Cat# 505829) at dilution of 1:300<br>anti-IL-17a antibody (BV605, Biolegend, Cat# 506927) at dilution of 1:300<br>anti-Foxp3 antibody (PE, eBioscience, Cat# 12-5773-82) at dilution of 1:300<br>anti-RORgt antibody (Alexa Fluor 647, BD Biosciences, Cat# 562682) at dilution of 1:300<br>isotype IgG (PE-Cy5, Biolegend, Cat# 400931) at dilution of 1:300<br>IgG2 lambda (Alexa Fluor 700, BD Biosciences, Cat# 557985) at dilution of 1:300<br>IgG2a (APC, BD Biosciences, Cat# 554690) at dilution of 1:300<br>IgG2a (PE-Cy7, Biolegend, Cat# 400521) at dilution of 1:300<br>IgG2b (BV650, BD Biosciences, Cat# 563233) at dilution of 1:300<br>IgG2a (BV605, Biolegend, Cat# 400539) at dilution of 1:300<br>IgG2b (Pacific Orange, BD Biosciences, Cat# 553989) at dilution of 1:300
Validation	Antibody validation can be found on the respective manufacturers websites, including relevant citations using these antibodies. In addition, we validated staining conditions of the antibodies (titer, temparature etc.) in our previous works (Nature Microbiology 2020 5 486; JCI 2019 130 3702; cells 2019 8 1121).

Animals and other organisms

Policy information about studies involving animals; ARRIVE guidelines recommended for reporting animal research

Laboratory animals	8-12 week-age male and female GF 129SvEv background IL-10-deficient mice (Il10-/-) were obtained from the University of North Carolina National Gnotobiotic Rodent Resource Center. Il10-eGFP-reporter (Il10+/eGFP) mice on a C57BL/6J background mice were originally provided by Dr. C. L. Karp (Global Health, Bill & Melinda Gates Foundation, USA) and raised in the National Gnotobiotic Rodent Resource Center. 8-12 week-age male and female Il10+/eGFP mice were used. Germ-free and gnotobiotic mice were maintained in positive-pressure isolators and housed in separate polycarbonate cages at constant room temperature (22°C ± 10%), air humidity (50% ± 20%), and a light/dark cycle of 12 h. Mice had free access to food and water. Standard mouse chow (TD2020SX; Teklad Diets, Madison, WI) was sterilized by irradiation at 25 kGy.
Wild animals	The study did not involve wild animals.
Field-collected samples	The study did not involve samples collected in the field.
Ethics oversight	All animal experiments were performed in compliance with all relevant ethical regulations for animal testing and research as described in the National Institutes of Health Guide for the Care and Use of Laboratory Animals. The study received ethical approval by the University of North Carolina - Chapel Hill Institutional Animal Care and Use Committee (18-266.0-B).

Note that full information on the approval of the study protocol must also be provided in the manuscript.

Human research participants

Policy information about studies involving human research participants

Population characteristics	The single fecal sample used for transplant into germ-free mice recipients was isolated from a healthy 42-year-old Asian male with no known diseases or current treatment. Genotyping was not performed
Recruitment	Volunteers were solicited from the local laboratory research community, specifying healthy status with no active inflammatory diseases. No monetary inducements were offered for participation. Participants were motivated to advance research, but no biases were evident.
Ethics oversight	The human fecal sample for transplant to mice was collected in compliance with all relevant ethical regulations for work with human participants, and with informed consent from the participant under the approved University of North Carolina Institutional Review Board protocol #17-1528.

Note that full information on the approval of the study protocol must also be provided in the manuscript.

Flow Cytometry

Plots

Confirm that:

☒ The axis labels state the marker and fluorochrome used (e.g. CD4-FITC).

☒ The axis scales are clearly visible. Include numbers along axes only for bottom left plot of group (a 'group' is an analysis of identical markers).

☒ All plots are contour plots with outliers or pseudocolor plots.

☒ A numerical value for number of cells or percentage (with statistics) is provided.

Methodology

Sample preparation	Single cells were stained for 20 min at 4°C after FcγRII/III blocking with anti-CD16/CD32 monoclonal antibody. For intracellular staining, cells were re-stimulated with 50 ng/ml phorbol 12-myristate 13-acetate (Sigma) and 500 ng/ml ionomycin (Sigma) for 4 h at room temperature with 1 μl/ml protein transport inhibitor (GolgiStop, BD) during the last 3 h. After washing, cells were first surface stained, then fixed for 5 min at 37°C using PBS containing 4% paraformaldehyde (Electron Microscopy Sciences) and 0.01% Tween20 (Fisher Scientific), permed using PBS containing 0.1% Triton X-100 (MP Biomedicals), 0.5% BSA (Sigma), 2 mM EDTA (Corning) for 45 min at room temperature, and stained overnight with indicated antibodies. Cells were washed and resuspended in PBS containing 1% bovine serum albumin (BSA) and then analyzed on a LSRII flow cytometer with FACSDiva software version 6.0 (BD Biosciences) and FlowJo software version 10 (FlowJo, OR, USA).
Instrument	LSRII flow cytometer (BD Biosciences, CA, USA)
Software	FACSDiva software version 6.0 (BD Biosciences)<br>FlowJo software version 10 (FlowJo, OR, USA)
Cell population abundance	For flow analysis, 12000-30000 of singlet live CD45+ cells were analyzed.
Gating strategy	Singlet live CD45+ cells were analyzed by FlowJo software version 10 (FlowJo, OR, USA) with the following gate strategy: B cell (B220+CD19+), CD4+ T cell (TCRβ+CD3+CD4+CD8neg), macrophage (TCRβnegCD11b+CD64+) and dendritic cell (TCRβnegCD64negMHCII+CD11c+). For GFP-positive gate, GFP-negative colonic lamina propria cells from C57BL/6 wild-type mice were stained with all antibodies used in the experiment as a fluorescence-minus-one control

☒ Tick this box to confirm that a figure exemplifying the gating strategy is provided in the Supplementary Information.
